# Supplementary material for: Characterization of some fungal pathogens causing anthracnose disease on yam in Cross River State, Nigeria
Source: PLoS One. 2022 Jun 29;17(6):e0270601. doi: 10.1371/journal.pone.0270601 (PMC9242479; doi:10.1371/journal.pone.0270601)
Supplement: S1 Table — (DOCX) [file pone.0270601.s001.docx]

**S1 Table.** **Primers sets used for identification of the fungal isolates.**

| Primer number | Primer name | Direction | Sequence |
| --- | --- | --- | --- |
| P1 | YamCgITS1 | F | CCAGCGGAGGGATCATTACT |
|  |  | R | AAGTTCAGCGGGTATTCCTACC |
| P2 | YamCgITS2 | F | CCTGCGGAGGGATCATTACT |
|  |  | R | AAGTTCAGCGGGTATTCCTACC |
| P3 | YamCgITS3 | F | CCTGCGGCGGGATCATTACT |
|  |  | R | AAGTTCAGCGGGTATTCCTACC |
