# Supplementary material for: Characterization of some fungal pathogens causing anthracnose disease on yam in Cross River State, Nigeria
Source: PLoS One. 2022 Jun 29;17(6):e0270601. doi: 10.1371/journal.pone.0270601 (PMC9242479; doi:10.1371/journal.pone.0270601)
Supplement: S2 Table — (DOCX) [file pone.0270601.s002.docx]

**S2 Table.** **Percentage nucleotide similarity among the fungal isolates from yam.**

| **Isolate/strain** | **KC010547** | **Ca5** | **Ca11** | **Ca14** | **Ca24** | **Ca32** | **Ca34** |
| --- | --- | --- | --- | --- | --- | --- | --- |
| KC010547* | 100 | 74.3 | 76.2 | 68.9 | 70.9 | 71.9 | 60.1 |
| Ca5 |  | 100 | 75.1 | 81.4 | 67.7 | 62.3 | 59.1 |
| Lt1 |  |  | 100 | 77.0 | 68.8 | 71.3 | 57.2 |
| Ca14 |  |  |  | 100 | 73.2 | 68.8 | 69.2 |
| Ca24 |  |  |  |  | 100 | 65.5 | 59.9 |
| Ca32 |  |  |  |  |  | 100 | 57.8 |
| Ca34 |  |  |  |  |  |  | 100 |

*Indicates the reference sequence from which the ITS primer 2 were designed
