# Supplementary material for: Characterization of some fungal pathogens causing anthracnose disease on yam in Cross River State, Nigeria
Source: PLoS One. 2022 Jun 29;17(6):e0270601. doi: 10.1371/journal.pone.0270601 (PMC9242479; doi:10.1371/journal.pone.0270601)
Supplement: S1 Raw images — (PDF) [file pone.0270601.s003.pdf]

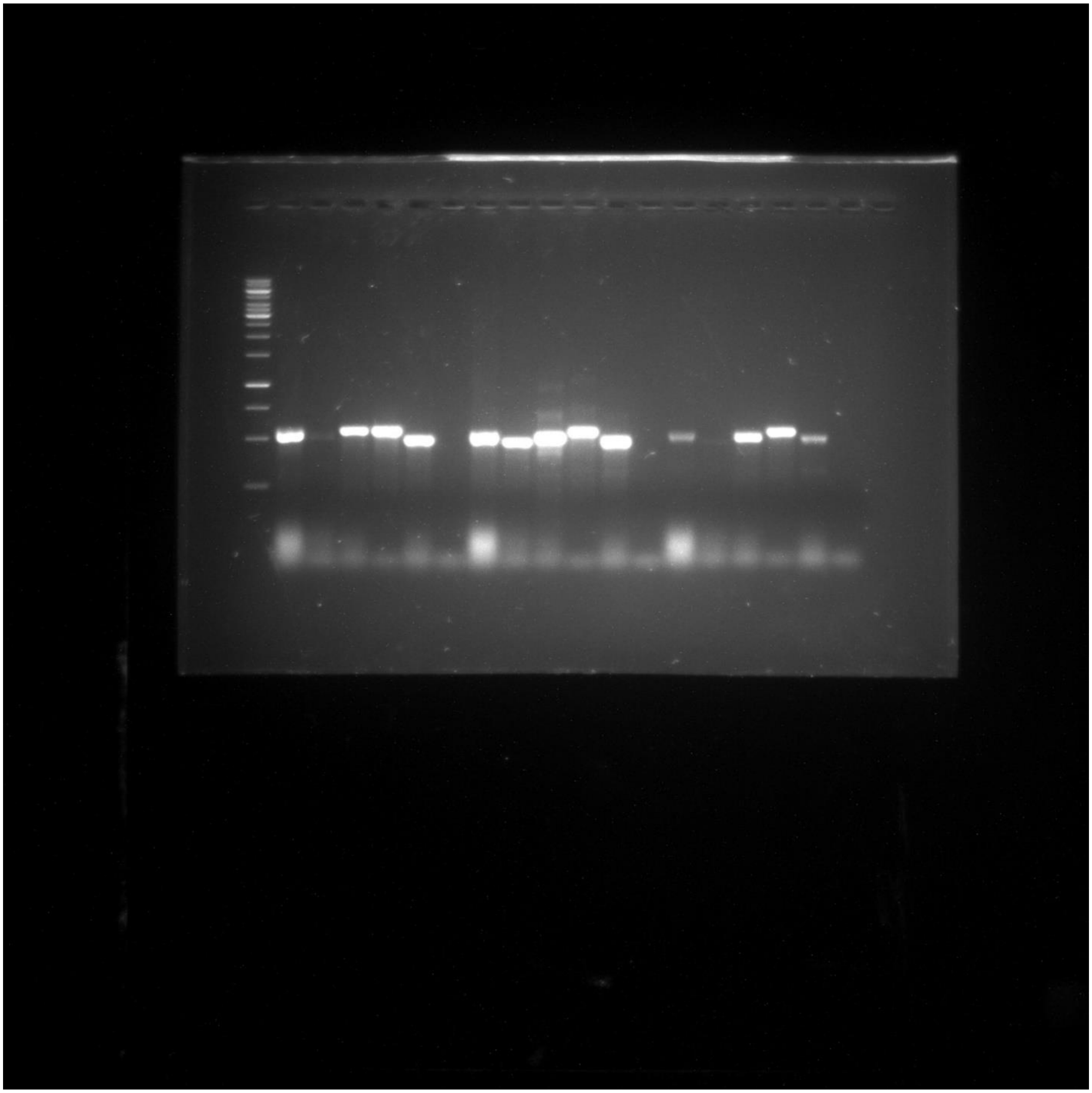

S1 Fig 1. PCR amplification of ITS gene from 8 isolates of *Colletotrichum alatae* using the three sets of primers (P1-P3).

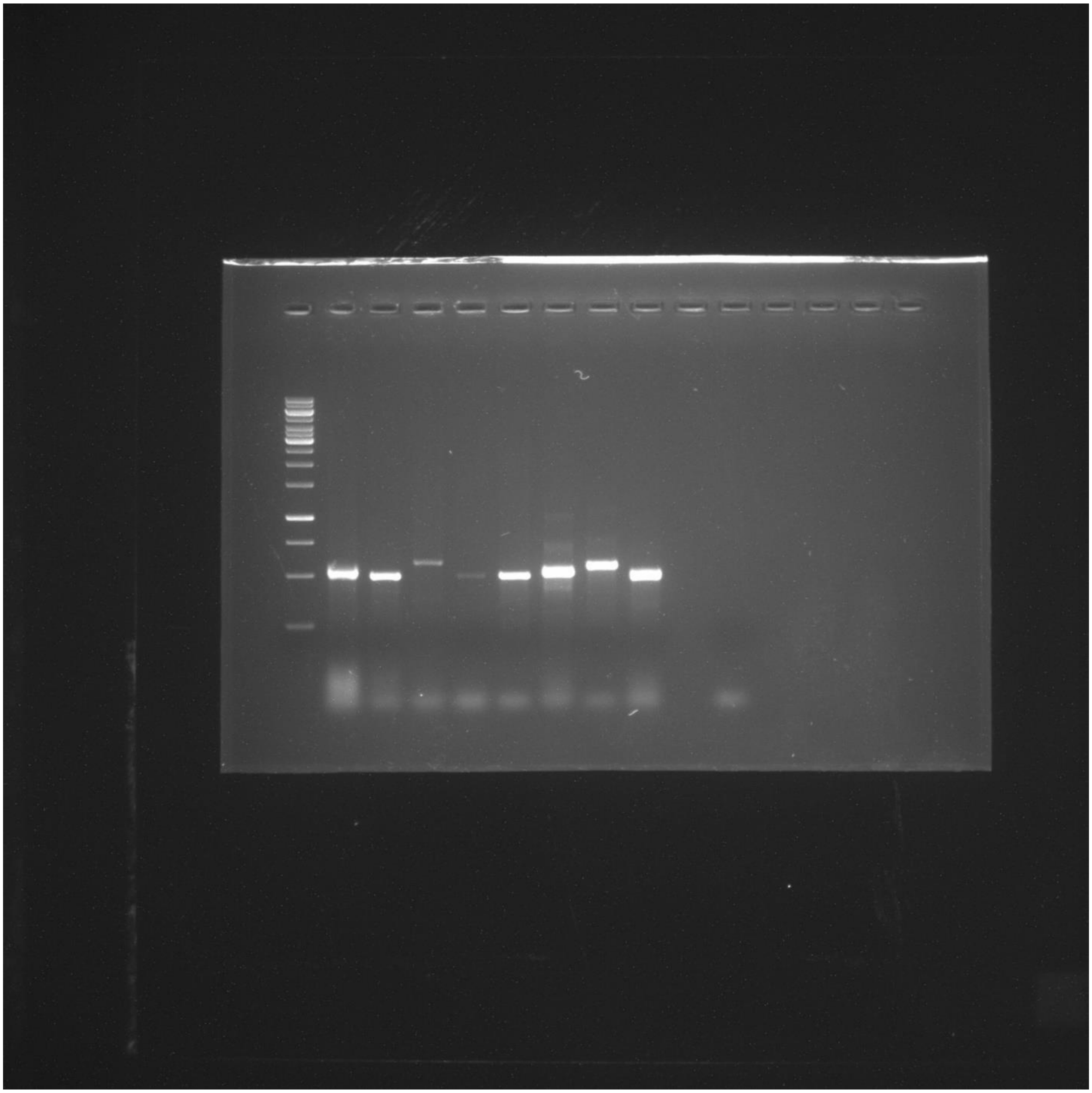

S1 Fig. 2. PCR amplification of ITS gene from 8 isolates of *Colletotrichum alatae* using primer set 2 (P2).
