# Supplementary material for: Characterization of some fungal pathogens causing anthracnose disease on yam in Cross River State, Nigeria
Source: PLoS One. 2022 Jun 29;17(6):e0270601. doi: 10.1371/journal.pone.0270601 (PMC9242479; doi:10.1371/journal.pone.0270601)
Supplement: Attachment — Submitted filename: PONE-D-21-40096_reviewed.doc [file pone.0270601.s004.doc]

**PLOS ONE**

**Characterization of Colletotrichum alatae causing yam anthracnose in Cross River State, Nigeria**

|  | --Manuscript Draft-- |  |
| --- | --- | --- |
|  |  |  |
| **Manuscript Number:** | PONE-D-21-40096 |  |
| **Article Type:** | Research Article |  |
|  |  | |
| **Full Title:** | Characterization of Colletotrichum alatae causing yam anthracnose in Cross River | |
|  | State, Nigeria |  |
| **Short Title:** | Characterization of Colletotrichum alatae in Cross River State | |
|  |  |  |
| **Corresponding Author:** | Valentine Otang Ntui, Ph.D |  |
|  | International Institute of Tropical Agriculture |  |
|  | Nairobi, Nairobi Municipality KENYA |  |
| **Keywords:** | Anthracnose; Colletotrichum alatae; Dioscorea spp.; | Fungal diseases; ITS; |
|  | Phylogeny; Yam |  |
|  |  |  |
| **Abstract:** | Yam anthracnose, caused by Colletotrichum alatae, | is the most serious fungal |
|  | disease affecting white and water yam production. Screening of available landraces for | |
|  | new sources of durable resistance to the pathogen are a continuous process. The | |
|  | pathogens causing anthracnose in Dioscorea alata and Dioscorea rotundata farms | |
|  | in Cross River State yam belt region were characterized. Diseased yam leaves with | |
|  | anthracnose symptoms collected from the farms were used in the isolation, purification | |
|  | and identification of Colletotrichum alatae strains using morphological, cultural, and | |
|  | molecular methods. Leaf chlorosis, leaf edge necrosis, blights, dark brown to black leaf | |
|  | spots, shot holes, necrotic vein banding and vein browning were the predominant | |
|  | symptoms observed. Eight isolates, Ca5, Ca11, Ca14, Ca16, Ca22, Ca24, Ca32 and | |
|  | Ca34 of C. alatae were obtained from the infected yam leaves, with Ca11 as the most | |
|  | prevalent, occurring in all the locations. Sequence analysis of the ITS region revealed | |
|  | <80% nucleotide identity between the isolates and the reference C. gloeosporioides , | |
|  | suggesting that the isolates are highly distinct. Pathogenicity test showed that all the | |
|  | isolates displayed typical symptoms of anthracnose disease as were observed in the | |
|  | field, but Ca11 was the most virulent. Inoculation of 20 D. alata and 13 D. rotundata | |
|  | landraces with isolate Ca11, revealed different degrees of resistance; 36.63% of the | |
|  | landraces were susceptible (S), 12.12% were highly susceptible (HS), 15.15% were | |
|  | moderately resistant (MS), 18.18% were resistant while 18.18% were highly resistant | |
|  | (HR). D. alata landraces were the most susceptible. This study revealed that | |
|  | anthracnose is prevalent and may assume epidemic dimension in the yam growing | |
|  | communities of the state. There is need for increased effort in the breeding of yam for | |
|  | anthracnose resistance. This will ensure productive yam farming and contribute | |
|  | substantially to food security and sustainable economic growth in these communities | |
|  | and the state, at large. |  |
| **Order of Authors:** | Nkese Ime Okon, MSc |  |
|  |  |  |
|  | Aniedi-Abasi Akpan Markson, Ph.D |  |
|  |  |  |
|  | Ekeng Ita Okon, MSc |  |
|  |  |  |
|  | Effiom Eyo Ita, Ph.D |  |
|  |  |  |
|  | Edak Aniedi Uyoh, Ph.D |  |
|  |  |  |
|  | Ene-Obong Effiom Ene-Obong, Ph.D |  |
|  |  |  |
|  | Valentine Otang Ntui, Ph.D |  |
|  |  |  |
| **Additional Information:** |  |  |
| **Question** | **Response** |  |
|  |  | |
| **Financial Disclosure** | This research was supported by the Nigerian Tertiary Education Trust Fund (TET Fund) | |
|  | grant TETFUND/DR&D/CE/NRF/2016/STI/13/VOL.1 awarded to the Department of | |
| Enter a financial disclosure statement that | Genetics and Biotechnology, University of Calabar, Calabar, Nigeria, for "Production of | |
|  |  |  |

Powered by Editorial Manager® and ProduXion Manager® from Aries Systems Corporation

| describes the sources of funding for the | | | | | | | | anthracnose-resistant yam seedlings for use by Nigerian farmers. |
| --- | --- | --- | --- | --- | --- | --- | --- | --- |
| work included in this submission. Review | | | | | | | |  |
| the [submission guidelines](http://journals.plos.org/plosone/s/submission-guidelines#loc-financial-disclosure-statement) for detailed | | | | | | | |  |
|  |  |  | |  |  |  |  |  |
| requirements. View published research | | | | | | | |  |
| articles from [*PLOS ONE*](http://journals.plos.org/plosone/) for specific | | | | | | | |  |
| examples. | |  | | |  | | |  |
| This statement is required for submission | | | | | | | |  |
| and **will appear in the published article** if | | | | | | | |  |
| the submission is accepted. Please make | | | | | | | |  |
| sure, it is accurate. | | | | | | | |  |
|  |  |  | |  |  |  |  |  |
| **Unfunded studies** | | | | | | | |  |
| Enter: *The author(s) received no specific* | | | | | | | |  |
| *funding for this work.* | | | | | | | |  |
| **Funded studies** | | | | | | | |  |
| Enter a statement with the following details: | | | | | | | |  |
| • Initials of the authors who received each | | | | | | | |  |
| award | | | | | | | |  |
| • Grant numbers awarded to each author | | | | | | | |  |
| • The full name of each funder | | | | | | | |  |
| • URL of each funder website | | | | | | | |  |
| • Did the sponsors or funders play any role in | | | | | | | |  |
| the study design, data collection and | | | | | | | |  |
| analysis, decision to publish, or preparation | | | | | | | |  |
| of the manuscript? | | | | | | | |  |
| **• NO** - Include this sentence at the end of | | | | | | | |  |
| your statement: *The funders had no role in* | | | | | | | |  |
| *study design, data collection and analysis,* | | | | | | | |  |
| *decision to publish, or preparation of the* | | | | | | | |  |
| *manuscript.* | | | | | | | |  |
| **• YES** - Specify the role(s) played. | | | | | | | |  |
| * Typeset | | | | | | | |  |
| **Competing Interests** | | | | | | | | The authors declare that no conflicts of interest exist |
| Use the instructions below to enter a | | | | | | | |  |
| competing interest statement for this | | | | | | | |  |
| submission. On behalf of all authors, | | | | | | | |  |
| disclose any [competing interests](http://journals.plos.org/plosone/s/competing-interests) that | | | | | | | |  |
|  | | |  | | | |  |  |
| could be perceived to bias this | | | | | | | |  |
| work—acknowledging all financial support | | | | | | | |  |
| and any other relevant financial or non- | | | | | | | |  |
| financial competing interests. | | | | | | | |  |
| This statement is required for submission | | | | | | | |  |
| and **will appear in the published article** if | | | | | | | |  |
| the submission is accepted. Please make | | | | | | | |  |
| sure, it is accurate and that any funding | | | | | | | |  |
|  |  |  | |  |  |  |  |  |

*Powered by Editorial Manager® and ProduXion Manager® from Aries Systems Corporation*

sources listed in your Funding Information

later in the submission form are also

declared in your Financial Disclosure

statement.

View published research articles from

[*PLOS ONE*](http://journals.plos.org/plosone/) for specific examples.

**NO authors have competing interests**

Enter: *The authors have declared that no*

*competing interests exist.*

**Authors with competing interests**

Enter competing interest details beginning

with this statement:

*I have read the journal's policy and the*

*authors of this manuscript have the following*

*competing interests: [insert competing*

*interests here]*

* Typeset

| **Ethics Statement** | Not field research |
| --- | --- |

Enter an ethics statement for this

submission. This statement is required if

the study involved:

- Human participants
- Human specimens or tissue
- Vertebrate animals or cephalopods
- Vertebrate embryos or tissues
- Field research

Write "N/A" if the submission does not

require an ethics statement.

General guidance is provided below.

Consult the [submission guidelines](http://journals.plos.org/plosone/s/submission-guidelines#loc-guidelines-for-specific-study-types) for

detailed instructions. **Make sure that all**

**information entered here is included in the**

**Methods section of the manuscript.**

*Powered by Editorial Manager® and ProduXion Manager® from Aries Systems Corporation*

**Format for specific study types**

**Human Subject Research (involving human**

**participants and/or tissue)**

- Give the name of the institutional review board or ethics committee that approved the study
- Include the approval number and/or a statement indicating approval of this research
- Indicate the form of consent obtained (written/oral) or the reason that consent was not obtained (e.g., the data were analyzed anonymously)

**Animal Research (involving vertebrate**

**animals, embryos, or tissues)**

- Provide the name of the Institutional Animal Care and Use Committee (IACUC) or other relevant ethics board that reviewed the study protocol, and indicate whether they approved this research or granted a formal waiver of ethical approval
- Include an approval number if one was obtained
- If the study involved *non-human primates*, add *additional details* about animal welfare and steps taken to ameliorate suffering
- If anaesthesia, euthanasia, or any kind of animal sacrifice is part of the study, include briefly which substances and/or methods were applied

**Field Research**

Include the following details if this study

involves the collection of plant, animal, or

other materials from a natural setting:

- Field permit number
- Name of the institution or relevant body that granted permission

| **Data Availability** | Yes - all data are fully available without restriction |
| --- | --- |

Authors are required to make all data

underlying the findings described fully

available, without restriction, and from the

time of publication. PLOS allows rare

exceptions to address legal and ethical

concerns. See the [PLOS Data Policy](http://journals.plos.org/plosone/s/data-availability) and

[FAQ](http://journals.plos.org/plosone/s/data-availability#loc-faqs-for-data-policy) for detailed information.

*Powered by Editorial Manager® and ProduXion Manager® from Aries Systems Corporation*

A Data Availability Statement describing

where the data can be found is required at

submission. Your answers to this question

constitute the Data Availability Statement

and **will be published in the article**, if

accepted.

**Important:** Stating ‘data available on request

from the author’ is not sufficient. If your data

are only available upon request, select ‘No’ for

the first question and explain your exceptional

situation in the text box.

Do the authors confirm that all data?

underlying the findings described in them

manuscript is fully available without

restriction?

**Describe where the data may be found in**	All relevant data are within the manuscript and its Supporting Information files.

**full sentences. If you are copying us**

**sample text, replace any instances of XXX**

**with the appropriate details.**

- If the data are **held or will be held in a public repository**, include URLs, accession numbers or DOIs. If this information will only be available after acceptance, indicate this by ticking the box below. For example: *All XXX files are available from the XXX database (accession number(s) XXX, XXX.*).
- If the data are all contained **within the manuscript and/or Supporting Information files**, enter the following: *All relevant data are within the manuscript and its Supporting Information files.*
- If neither of these applies but you can provide **details of access elsewhere**, with or without limitations, please do so. For example:

*Data cannot be shared publicly because of [XXX]. Data are available from the XXX Institutional Data Access / Ethics Committee (contact via XXX) for researchers who meet the criteria for access to confidential data.*

*The data underlying the results*

*presented in the study are available*

*from (include the name of the third party*

*Powered by Editorial Manager® and ProduXion Manager® from Aries Systems Corporation*

*and contact information or URL).*

- This text is appropriate if the data are owned by a third party and authors do not have permission to share the data.

* Typeset

Additional data availability information:

*Powered by Editorial Manager® and ProduXion Manager® from Aries Systems Corporation*

Manuscript

[Click here to access/download;Manuscript;Pathology](https://www.editorialmanager.com/pone/download.aspx?id=30352691&guid=047d6bda-22c5-4fc6-9e86-266806a71fd2&scheme=1)

[manuscript.docx](https://www.editorialmanager.com/pone/download.aspx?id=30352691&guid=047d6bda-22c5-4fc6-9e86-266806a71fd2&scheme=1)

**Characterization of *Colletotrichum alatae* causing yam anthracnose in Cross River State, Nigeria**

**Short Title: Characterization of *Colletotrichum alatae* in Cross River State**

**Nkese Ime Okon^1^, Aniedi-Abasi Akpan Markson^2^, Ekeng Ita Okon^2^, Effiom Eyo Ita^1^, Edak Aniedi Uyoh ^1^, Ene-Obong Effiom Ene-Obong^1^, Valentine Otang Ntui ^1,3^***

^1^Department of Genetics and Biotechnology, University of Calabar, Calabar. P. M. B. 1115, Calabar.

^2^Department of Plant and Ecological Studies, University of Calabar, Calabar. P. M. B. 1115, Calabar.

^3^International Institute of Tropical Agriculture, Nairobi, Kenya

*****Correspondence:** [**v.ntui@cgiar.org**](mailto:v.ntui@cgiar.org)**;** [**ntuival@yahoo.com**](mailto:ntuival@yahoo.com)

**ORCID**

**Ntui: 0000-0003-4709-8087**

**Abstract**

*m*

and may assume an epidemic dimension in the yam growing communities of the state. There is a need for increased effort in the breeding of yam for anthracnose resistance. This will ensure productive yam farming and contribute substantially to food security and sustainable economic growth in these communities and the state, at large.

**Keywords:** Anthracnose; *Colletotrichum alatae*; *Dioscorea* spp.; Fungal diseases; ITS; Phylogeny; Yam

**Introduction**

Yams (*Dioscorea* spp.) are monocotyledonous plants with underground tubers and constitute the predominant starchy staple in sub-Saharan Africa especially in the five West African countries (Nigeria, Cote d’Ivoire, Ghana, Benin, Togo) widely regarded as the ‘Yam belt region’ [1, 2]. There are about 600 known species of yam, of these, only 11 are edible, and six out of these are cultivated and consumed in Nigeria [2, 3]. Among these six species, white yam (*Dioscorea rotundata*) commands the highest market value owing to the superiority and preference of its tubers for food in Nigeria [4]. Nigeria has been consistent in the lead of yam producing countries worldwide. Available statistics indicates that in 2012, Nigeria accounted for over 65% (38 million tons) of the over 58. 8 million tons of yams produced, valued at $7.75 billion and cultivated on 2.9 million hectares [5]. Recently, Nigeria’s annual yam production statistics stands at over 45,004 million metric tons [6].

Except for cereals and cassava, yam is the most important food crop in Nigeria. Yam contributes more than 200 dietary calories per day for over 50% (100 million) of the Nigerian population. It is the only crop which is usually celebrated during and after harvest [6]. Yam is usually eaten directly with sauce after boiling, fried in oil, or roasted. It is also boiled and pounded into a thick dough popularly called pounded yam and eaten with soup. In the Western part of Nigeria, a reconstituted form called ‘amala’ (another popular dough) is the people’s favourite. The medicinal value of yam is a function of its diosgenin content. Diosgenin (a kind of sapogenin) is a prominent source of hemi synthesis of birth control pills (with progesterone and estrogen as well as similar hormones) and corticosteroids [7].

Despite the increasing yam production figures reported for Nigeria [5, 6, 8], yam production is generally constrained by a myriad of problems some of which are weed pressure, decline in soil fertility, storage pests, high cost of labour, cost of land preparation and maintenance, staking and barn making and, most importantly, field pests and diseases. Among the diseases plaguing yam, anthracnose or die back disease is one of the most ravaging [2, 9]. It is one of the major constraints limiting white yam (*D. rotundata*) and water yam (*D. alata*) production in the tropics [2, 10]. In Nigeria, *Colletotrichum* disease complex (commonly referred to as anthracnose or die-back) remains one of the most challenging and destructive diseases, causing heavy losses in yam [11]. Anthracnose has been implicated in yam tuber yield losses ranging from 50 to 90% under favourable conditions for pathogen infection, establishment, and disease development [9]. Infection and disease symptoms are concentrated on leaves, though yam petioles, stems, and tubers are also known to be infected. The capability of *Colletotrichum* for multiple routes of transmission and its ability to persist in the field are the major strengths of this pathogen complex to cause extensive crop damage. It is capable of being transmitted from foliage to tuber, and from tuber to foliage in the following season [11] while overwintering in infected planting material (stored tubers). Alternative hosts and crop debris constitute the main sources of pathogen inoculum [13,14].

Reports on extensive survey of anthracnose disease incidence and severity on yam caused by C*olletotrichum* species in Nigeria are scanty, especially, in the South-South geopolitical zone of the country. However, a 61.7% occurrence of C*olletotrichum* species among seven other targeted pathogens and an 84.2% occurrence of *Colletotrichum* spp. among species of *Colletotrichum* isolated were reported in the southern guinea savanna and the southern forest area of Nigeria [9]. Symptoms vary based on yam species and ecological region of occurrence [11]. Typical symptoms exhibited by most *Dioscorea alata* cultivars grown in the Southern Guinea savanna region are circular black spots on leaf surfaces, expanding to express leaf edge necrosis and then progressing to vine blackening and tip die-back. On the other hand, typical symptoms reported for *D. rotundata*, are black circular spots, randomly distributed on leaf surfaces with extensive defoliation and vine blackening from severe infection. In the southern forest region, symptoms on *D. alata* were said to appear mostly as streak

browning lesions starting off on leaf veins and rapidly expanding to cover the entire leaf [11]. Comparatively, impact of anthracnose disease on yam is often rated higher than that of viruses. A 72% disease severity was reported for anthracnose in six genotypes of *D. alata* in Ibadan, Western Nigeria over that of some viruses under similar test conditions [15]. Also, an extensive assessment of field occurrence of *C. gleosporoides* in Benue state has been reported [16].

Cross River is one of the major yams producing states in Nigeria. In the present study, the prevalence and distribution of yam anthracnose in this state as well as proper characterization and identification of the anthracnose isolates obtained across the state were carried out. Commonly cultivated landraces/accessions of *D. alata* and *D. rotundata* in Cross River state were also screened for resistance against a virulent strain of the pathogen isolated from the infected samples. Results obtained should help proffer solutions on how to manage the disease.

**Materials and methods**

**Study area**

The study area constitutes the three senatorial districts of Cross River state covering the southern guinea savanna region to the north and the humid rain forest agro-ecological zone to the south. The northern part of the state where yam-producing communities like Ogoja, Yala and Bekwara are located lies within the southern guinea savanna region while the southern part of the state (Obubra, Yakurr, Ikom, Akpabuyo and Calabar South) is in the humid rainforest agro-ecological zone (Fig.1). Cross River has an annual temperature fluctuating between 22^o^C and 32^o^C and annual rainfall of over 2000

mm and is located within longitude 4° 57' 0" North and latitude 8° 19' 0" East. Cross River State is agrarian with a greater population of her inhabitants predominantly engaging in farming, particularly, yam, cassava, banana, and plantain cultivation.

**Collection of infected yam leaves from sampling locations**

A total of 21 locations were sampled in six main areas across the three senatorial districts. Villages engaged in commercial production of yam were selected for the survey. In each village about 5 to 6 farms were visited. The farms were surveyed for *D. alata* and *D*. *rotundata leaves* with die back and necrotic lesions symptoms. Infected leaf samples were collected, labeled, and packaged accordingly for subsequent laboratory studies. Sampled areas and farm locations are presented in Table 1.

**Collection of yam landraces from sampling locations**

Yam Landraces of *D. alata.* L and *D. rotundata* Poir were collected in September 2019 from local farmers in Cross River State and Benue State. Yam accessions were also obtained from National Root Crop Research Institute (NRCRI), Umudike, Abia State, Nigeria. A total of 20 *D. alata* and 13 *D. rotundata* Landraces/accessions were collected (Table 2). Each yam tuber was packaged separately in a plastic bag and labelled accordingly. The tubers were stored in a well-ventilated room to break dormancy. Once sprouting was noticed, the tubers were cut into sets of average weight 350 g [17]. The cut setts were treated with wood ash before planting.

**Isolation and identification of isolates from infected water yam leaves using cultural and morphological characteristics**

The symptomatic yam leaves were surface sterilized with 0.1% sodium hypochlorite for three minutes and 70% alcohol for one minute. The leaves were rinsed three times in changes of sterile distilled water. Four pieces (5mm diameter) of each infected leaf tissues were cut with flame-sterilized scalpel and inoculated on the solidified potato dextrose agar (PDA) medium in different plates. The inoculated plates were stored at room temperature (28℃) and observations were made daily for emergence of colonies. Sub-culturing was done weekly to obtain pure cultures of the isolates. The isolates were stored on slants of potato dextrose agar (PDA) in properly corked 250 ml conical flasks. Subcultures were made in petri dishes using PDA when the need arose. The structures of the isolates were identified using standard reference atlas of imperfect fungi by Barnett and Hunter [18] and with literature on identification of *Colletotrichum* species by Abang et al. [19].

**Identification of isolates using molecular methods**

**DNA extraction**

DNA extraction from the fungal mycelia was done using a modified CTAB protocol [20]. The mycelia were harvested by filtration through mesh sieves (40 µL), washed with sterile distilled water, placed on Whatman filter paper to remove excess water and ground to a fine paste in 400 µL of extraction buffer using mortar and pestle. The paste was then put in microfuge tubes and incubated in a water bath at 65°C for 15 min, followed by centrifugation at 12000 rpm for 5 min. 400 µL of supernatant was transferred into new eppendorf tubes and 250 µL of chloroform: Iso amyl-alcohol (24:1, v/v) added to each tube, mixed by inversion, and centrifuged at 13,000 rpm for 10 min. The upper aqueous phase was transferred into a clean microcentrifuge tube and 50 µL of 7.5 M Ammonium Acetate, followed by 400 µL of ice-cold ethanol were added to each tube to precipitate the DNA. This was then mixed by slow inverted movements that caused the DNA to precipitate at the bottom of the tubes. The tube containing the DNA was centrifuged at 13,000 rpm for 5 min followed by decantation. The DNA was washed twice with 0.5 mL of 70% ethanol by centrifuging at 15,000 rpm for 5 min. The DNA was then dried and 50 µL of TE buffer added to dissolve it, before storage at - 20°C until required.

**PCR amplification of ITS-rRNA genes**

Nucleotide sequences for Internal Transcribed Spacer (ITS)-ribosomal RNA (rRNA) genes of *C. gloeosporioides* were downloaded from NCBI and aligned to identify conserved regions. As there were no conserved regions, three pairs of primers flanking the ITS1-ITS2 regions were designed from three different strains of *C. gloeosporioides.* Primer 1 was designed from *C. gloeosporioides* strain E6 with accession number KT325567.1. Primer 2 was designed from *C. gloeosporioides* strain C16 with accession number KC010547.1. Primer 3 was designed from *C. gloeosporioides* strain D1 accession number KT325559.1. The primer sequences (Table 3) were sent to Inqaba for synthesis. PCR was done in a 20µl reaction volume made up of 14.5µl distilled water, 2 µl of 10 x PCR buffer, 0.4µl of dNTP mix, 0.5µl each of 10 mM forward and reverse primer, 0.1µl of Hot start Taq polymerase and 2µl of

DNA. PCR amplifications were performed using the following conditions: initial denaturation at 95°C for 30seconds followed by 35 cycles of denaturation at 94°C for 30 seconds, annealing at 55°C for 30 seconds, extension at 72°C for 1 minute and a final extension at 72°C for 7 minutes. A total of 8 isolates were used for PCR analysis.

**Sequencing of the ITS region**

The PCR products were purified with QIAquick PCR purification Kit (Qiagen) according to the instructions in the user manual. The purified products were used to make PCR sequencing reaction. The sequencing reaction mixture consisted of 2.5 µL PCR product, 1.5 µL of 5X sequencing buffer, 0.5 µL of Big Dye Terminator, 0.5 µL of 10 mM of either YamCgITS2_F or YamCgITS2_R primer and 5µL of nuclease free water. PCR amplification was performed in a thermal cycler with initial denaturation step at 96˚C for 2 min followed by 40 cycles of denaturation at 96˚C for 10 s, annealing at 50 ˚C for 10 s, extension at 60˚C for 4 min, and final extension at 72˚C for 4 min. After PCR amplification, the sequencing product was purified by adding 50 µL of 100% ethanol, 2 µL of 3 M Sodium Acetate and 2 µL of 125 mM EDTA, and incubated at room temperature for 30 min. The mixture was centrifuged at 4000 rpm for 25 min at 20 ˚C. The pellets were washed with 70% ethanol and air dried for 20 min in the hood. The pellets were then resuspended in 10 µL Hi-Di formamide and incubated at 65 ˚C for 5 min, 95 ˚C for 2 min and cooled in ice. The samples were then sequenced using ABI 3130 DNA sequencer (Applied Biosystems, California, USA). The sequences were assembled, edited, and analyzed using Snap Gene software [(WWW.snapgene.com)](http://www.snapgene.com/). For each isolate, 4 replicates each for forward and reverse primers were sequenced.

**Phylogenetic analyses**

The nucleotide sequences for each isolate were used in BLASTn searches against the GenBank database [(http://www.ncbi.nlm.nih.gov/BLAST)](http://www.ncbi.nlm.nih.gov/BLAST) to identify the most similar sequences available in the database. There were high variations in the nucleotide identities therefore sequences in the GenBank that showed the highest similarity (>60%) to the isolates were used for alignment. Sequences

were aligned using the MUSCLE program and used subsequently for phylogenetic analyses based on the Maximum Likelihood method with 1000 bootstrapping. *C. gloeosporioides* isolate with accession number KC010547, from which the primers were designed was included in the analysis.

**Pathogenicity test (Koch’s Postulate)**

The pathogenicity of isolates from the infected water yam leaves was tested. Disease-free *D. alata* and *D. rotundata* yam tubers were planted in pots containing sterilized soils in the screen house maintained at normal ambient environmental temperature. Two months old leaves were dusted, rubbed gently with carborundum and sprayed with inoculums using a 450 mL atomizer spray gun. The inoculum was prepared using a 10-day-old pure cultures of *C. alatae* which was prepared and stored. The Petri dish containing the pure culture of the pathogen was flooded with distilled water and a sterile blade was used to scrape the mycelia off the solid medium to release the conidia into a 200 mL sterile beaker. The mixture was vigorously shaken using a magnetic shaker before filtering it through sterile cheesecloth to obtain spore (conidia) suspension. Spore (conidia) load of 1 x10^6^ per mL was prepared through serial dilutions and spore count was done using haemocytometer [21]. Each inoculated leaf was covered with transparent polythene bag for 24 hours. The set up was monitored every day for symptom expression.

**Screening of yam for tolerance to C. alatae: Whole plant bioassay**

The 33 yam landraces or accessions, three triplicates each, were screened in the screenhouse for anthracnose resistance. The yam plants were challenged with isolate Ca11 which was the most virulent according to the pathogenicity test. Whole plant inoculation was done according to the method of Kolade et al*.* [21] with some modifications. Two months after planting, five fully opened leaves were inoculated by dusting and gently rubbing with carborandum. The wounded region was rinsed by spraying with distilled water using a 450 ml plastic atomiser spray gun. The leaves were then dipped in the inoculum, prepared as above, for 5 minutes. Each inoculated leaf was covered with moistened polythene bags for 24 hrs to aid disease development [22]. Thereafter, the leaves were opened and observed for symptom development every alternate day, but data were recorded at 16 weeks’ postinoculation (WPI) [23]. The percentage of the leaf area exhibiting lesions was estimated for each accession and used for classification into various tolerance levels. This was done using a five-scale range where 0= no infection, 1= 1-20%, 2= 21-40%, 3= 41-60%, 4= 61-80% and 5= 81 -100% infection. The experiment was laid out in Completely Randomised Design (CRD) with. Disease incidence (DI%) per plant was computed using the formula:

DI (%) per stand = No. of leaves infected x 100

Total number of leaves	1

**Results**

**Symptoms and distribution of *C. alatae***

*C. alatae* symptoms were observed in all sites surveyed indicating the disease is widely distributed across the three senatorial districts. However, the symptoms were more prevalent in the central region, particularly, Yakurr compared to the other regions. Assessment of infected water yam stands in the sampled farms revealed a wide range of symptoms (Fig. 2). The symptoms varied from leaf tissue necrosis to shoots die-back, appearing as extensive scotch or blight, progressing from leaf margins towards the centre of the leaf lamina. Also, some symptoms appeared as yellow margins surrounding dark brown to black necrotic leaf tissues. Some expanding leaves were observed to be twisted, especially, at the apex. Some water yam stands exhibited leaf chlorosis and stunted growth. In Obubra sampling locations, symptoms include chlorosis (a), dark brown spot dotting the leaf lamina (b), enlarged white spot encircled by brownish ring (c), brown necrotic tissues affecting the leaf base around the petiole attachment portion (d). The predominant symptoms (e-l) in Yakurr area include, necrotic vein banding (e), brownish spot rimmed by yellow ring coalescing to form enlarged necrotic portions (g), chlorosis and blight encroaching from the margin towards the centre of the leaf (h), vein browning and leaf edge necrosis (i), necrosis of tissues resulting in large shot holes (j), chlorosis with leaf browning (k), leaf tissue bleaching causing twisted leaf apex (l). In Ogoja, the prevailing symptoms were chlorosis and blight encroaching from the margin towards the centre of the leaf (m),

large shot hole encircled by brown necrotic ring rimmed by a yellow ring (n), brownish ring surrounding light brown to white central portion (p), enlarged white necrotic ring (q), leaves dotted with shot holes resulting from falling off necrotic central portions (r), small brownish spot coalescing to form large brown necrotic areas on the leaves (s). The predominant symptoms displayed on leaves sampled from Akpabuyo were extensive blight and chlorosis (t), brownish to black spot merging into larger necrotic patches (u). In Calabar South, infected leaves were observed to exhibit leaf edge necrosis (v) and extensive blight, chlorosis and tissue necrosis advancing from leaf margin towards the centre, often from one half of the leaf lamina

**Identification of isolates from infected water yam leaves using cultural and morphological methods**

Following isolations from the sample locations, eight (8) isolates (Ca5, Cg11, Ca14, Ca16, Ca22, Ca24, Ca32 and Ca34) were obtained (Fig. 3) and described based on the cultural and morphological characteristics (Table 4). Observation of the pure cultures of the eight isolates, revealed striking culture characteristics of the mycelia in the growth medium. The characters described were mycelia colour, growth pattern, nature of mycelia, colour on the reverse side of the plate and growth rate (Table 4). Ca5, Ca16 Ca24 and Ca34, had white mycelia which gradually turned grey with age while Ca11, Ca14 and Ca32, had mycelial colours of orange, pink and cream, respectively. All, except Ca16 exhibited radial and circular growth patterns with concentric rings while all the isolates displayed cottony mycelial growth except Ca16 and Ca32. Based on the growth characteristics and literature, these isolates were confirmed to be *C. alatae* and had four forms which included the slow-growing grey (SGG), the fast-growing grey (FGG), the fast-growing salmon (FGS), and the fast-growing olive (FGO) forms (Table 4). Cylindrical shaped conidia with rounded ends were recorded for some of the isolates (Fig. 4). The mycelial growth rate per day for the various isolates ranged from 1.69 to 3.92 mm and were within the growth rate range of 3.6 to 11.2 mm recorded for *C. gloeosporioides* [24, 25].

**PCR amplification**

First, we selected 5 isolates and subjected them to PCR analysis using the three primer pairs shown in Table 3. Only primer 2 (YamITS2_F and YamITS2_R) amplified sequences of the ITS regions, including the 5·8S and 28S ribosomal RNA genes in all the isolates (Fig. 5a). Primers 1 and 3 did not amplify isolate Ca11, therefore, we used primer 2 for amplification of the ITS1-ITS2 regions, in all the remaining isolates. PCR fragments of approximately 500 bp were obtained (Fig. 5b). This result indicates that the isolated fungi are *Colletotrichum* spp., the same causative organism responsible for the manifestation of anthracnose symptoms in the leaf samples*.*

**Sequence analysis of the ITS region**

PCR products obtained using primer 2 were purified and sequenced. The nucleotide sequences of the ITS region for each isolate are presented in Table 5. To determine whether the isolates are different, we checked the nucleotide identity between the isolates and with the reference sequence using EMBOSS matcher-Pairwise Sequence Alignment [(https://www.ebi.ac.uk/Tools/psa/emboss_matcher/)](../(https://www.ebi.ac.uk/Tools/psa/emboss_matcher/)). Alignment of the ITS regions revealed nucleotide sequence identities ranging from 60.1 to 76.2% between the isolates and the reference sequence (Table 6). The *C. alatae* isolates share <82% nucleotide identity (Table 6) indicating that the isolates are equidistant and independent.

Next, we compared the nucleotide sequences with those of published ITS sequences of several C. *gloeosporioides* including the reference isolates. The evolutionary history was inferred using the UPGMA method. The percentage of replicate trees in which the associated taxa clustered together in the bootstrap test (1000 replicates) are shown next to the branches. The evolutionary distances were computed using the Maximum Composite Likelihood method and are in the units of the number of base substitutions per site. This analysis involved 22 nucleotide sequences. Codon positions included were 1st+2nd+3rd+Noncoding. All ambiguous positions were removed for each sequence pair (pairwise deletion option). Nucleotide sequences of *C. alatae* isolates were clearly distinguishable from C. *gloeosporioides* isolates (Fig. 6). Isolate Ca5 and Ca14 were found in the same cluster with 81.4% nucleotide identity. Isolate Ca34 is an out-group with <70% identity with other *C. alatae*

isolates but belong to the same cluster with C. *gloeosporioides* isolate KU097215. None of the *C. alatae* isolates was in the same cluster with the reference sequence indicating that the isolates are distinct.

**Pathogenicity test (Koch’s Postulate)**

The 8 isolates were subjected to pathogenicity test using susceptible water yam accessions. All the isolates displayed typical symptoms of anthracnose disease as were observed in the field. Anthracnose disease symptoms were not observed on the negative control leaves. However, TDA1100193 was the most resistant, displaying insignificant level of susceptibility. On re-isolation, they all exhibited patterns of growth as observed in the original isolates. However, based on the prevalence of the pathogen in all sample locations, virulence of the pathogen and severity of infection during pathogenicity test, Ca11 was selected and used as the test pathogen to screen for resistance against all the collected landraces/accessions.

**Screening *D. alata* and *D. rotundata* for resistance to *C. alatae* in the screen house**

All the twenty *D. alata* yam landraces and accessions screened for resistance against anthracnose pathogen *C. alatae* (Ca11) exhibited varying levels of severity of infection (Table 7). TDA1100193 with a disease severity score of 1 was the only accession that was highly resistant (HR) to infection by *C. alatae* representing only 5%*.* TDa 1100010 and CA14 with disease severity scores of 2 were considered as resistant to the *C. alatae.* On the other hand, TDA 1100154, TDa 1100432 CA5, CA6, CA11, CA13, CA15, CA16 and CA18 were found to be susceptible to the pathogen and the landraces CA7, CA9 and NA17 were highly susceptible to the anthracnose strain (Table 7). In the resistant and highly resistant landraces/accessions only 20% and 30% leaves were infected, respectively and symptoms were only visible 9 weeks after inoculation.

In *D*. *rot*undata, disease incidence ranged from 9% in BR6 to 95% in ER7 (Table 8). Among the 13 Landraces and accessions, 5, ER3, ER5, ER6, TDr100006 and CR12 were found to be highly resistant to the anthracnose strain used while CR1, ER2, ER4, and CR11 were resistant. ER7 was highly

susceptible while CR8 and NR9 were susceptible. The disease severity score ranged from 1 to 5 (Table 8).

**Discussion**

About 7 types of spots and 4 types of blights have generally been identified as symptoms of anthracnose disease on yam leaves [26]. These symptoms are said to be in the form of dark lesions which are usually surrounded with yellow halo. Some are reported to be seen as dark brown rings surrounding a light brownish necrotic portion. In the present study, the areas under survey presented a myriad of symptoms including the ones reported above. Other symptoms of anthracnose commonly expressed in white yam and water yam include leaf necrosis and dieback of vines [27]. The type of symptoms in a particular area seems to be substantially influenced by the prevailing ecological indices of the area. Reports of streak browning lesions starting off on leaf veins and rapidly expanding to cover the entire leaf was documented for *D. alata* in the forest region of Nigeria [11]. Here, necrotic vein banding, vein browning and leaf edge necrosis were common in Yakurr and Obubra (forest region). However, in Akpabuyo and Calabar South (also, in the forest zone) chlorosis and blight affecting the entire leaf margins progressing inwards were commonly observed. The contrasting nature of symptoms observed on the yams in the southern region of Calabar and Akpabuyo with those in earlier reports may be due to varietal differences in the yams under study. In the Southern Guinea savannas, symptoms were reported to commence with circular black spots on leaf surfaces and expanding to manifest as leaf edge necrosis and then progressing to vine blackening and tip die-back. Similar symptoms were commonly encountered in this study in Ogoja and Ikom, which fall within the same region. In addition, commonly displayed symptoms on infected water yam leaves included small brownish spots coalescing to form large brown necrotic areas on the leaves as well as large shot holes encircled by brown necrotic rings rimmed by yellow rings.

A comparison of the prevalence across the regions sampled showed that the frequency of the disease was principally based on the level of the cultivation of susceptible accessions. Based on this, Yakurr was the region that had the highest prevalence. Yakurr is known to be the highest producer of water

yam in Cross River state, and its location falls within the humid rainforest agro-ecological zone with an annual temperature fluctuating between 22^o^C and 32^o^C and annual rainfall of over 2000 mm reported to favour anthracnose disease development [11]. These are the likely reasons for the disease severity level recorded in this area.

Isolates of *C. alatae* are highly variable, and manifest a range of colony colours, growth rates and morphology [19, 28-32]. Three of the isolates in the present study bear similar morphological characteristics and exhibited comparable growth rates to two morphological forms described by [19]. Isolates Ca16 and Ca24 were comparable with the fast-growing grey (FGG) and Ca32 like fast-growing salmon (FGS). Several researchers have reported varying cultural characteristics ranging from mycelia colour, growth pattern, growth rate, nature of mycelia, colour changes with progressive growth and colour in media. Colour variation of isolates have been reported from normal white to light grey, greyish brown, grayish white, greenish grey, pinkish, and pinkish brown [29]. Martínez et al. [31] reported some isolates of *C. gloeosporioides* with varying cultural descriptions. They reported mycelia colours such as white, grey, or orange with cottony mycelium being dominant as well as luxuriant orange conidial masses with grey or white bottom color. Zipporah et al. [30] documented changes in colour of media in which isolates of *C. gloeosporioides* varied from pink, pale pink, pale orange to dark brown*.* Mycelial pigmentation varying from greenish-grey, dark, pinkish to milky white colour was reported by Fokunang et al. [33] to be exhibited by forms of *C. gloeosporioides* isolated from cassava stems showing dark brown lesions and deep cankers in Nigeria. They also observed that the isolates exhibited circular uniform or radial (mycelia growing in a ring-like pattern) growth pattern. Yee and Sariah [32] described four groups of *C. gloeosporioides* based on colour changes and growth pattern. The first three groups constituted forms with white mycelia which turned grey, olivaceous and greyish white respectively, with progress in growth while the fourth one was said to be smoky white with thick floccose aerial mycelium and orange conidial pustules at the centre. Kwodaga et al. [28] described six forms of *C. gloeosporioides* isolated from white yam (*D. rotundata*) having whitish and pinkish cottony mycelia growing radially with concentric rings. All the isolates obtained from infected

yam in this study share these characters. Isolates Ca5, Ca16, Ca24 and Ca34 had whitish mycelia which gradually turned grey with age. All, except Ca16 exhibited radial and circular growth patterns with concentric rings while all the isolates displayed cottony mycelial growth except Ca16 and CA32. Colours in the range of creamy, orange and pink were exhibited by Ca4, Ca14 and Ca32.

Anthracnose disease is said to be caused by *Colletotrichum* disease complex [34]. Though the first report of anthracnose on water yam in Nigeria in 1980 by Nwankiti and Okpala [35] was credited to *C. gloeosporioides*, it is however known today that the disease is most often caused by a complex of seemingly similar forms of *Colletotrichum*. This creates a challenge of identifying specifically, the cause of a particular infection in time. The systematics of the species complex of the genus, *Colletotrichum* since it was first reported has over the years been evolving based on the taxonomic tool employed by various scientists to characterize this group of fungi. *Colletotrichum* is the only genus of the family, Glomerellaceae and consists of some saprobes and endophytes [36] but dominated by pathogens causing diseases of virtually all categories of plant forms from fruits and vegetables [37-39] through cereals (grasses), pulses to root and tuber crops [11, 19, 40-42] in both tropical and temperate

[43] regions of the world. Owing to the multiplicity of forms and the host-specific nature of this group of organisms, there has been a lot of erroneous categorization, misidentification, and naming.

To edge over these challenges, some researchers have resorted to using various specific morphological (taxonomic) characters to classify species in the genus and have come up with what is referred to as

‘Accepted species. Sutton [44] came up with 39 accepted species based on morphological and cultural characteristics. Hyde et al. [45] published a more detailed work on this genus and categorized the forms into 66 names of commonly used and 19 names of uncertain taxonomic status. Although such efforts have made impressive contribution towards arriving at a more unambiguous and discrete characterizations of *Colletotrichum,* huge gaps are still eminent. Winch et al. [46] and Abang et al.

[19] reported that *Colletotrichum* isolates from diseased yam leaves were morphologically and genetically distinct but used a wide species concept to lump all yam isolates together under the name

*C. gloeosporioides*. Weir et al. [34] found that yam anthracnose isolates from Nigeria, along with those

from Barbados, Guadeloupe, and India, belonged to the same clade and matched the Slow-growing grey (SGG) group described by Abang et al. [19], and were thus classified as *C. alatae*. In Danzhou City, Hainan Province, China, anthracnose-like lesions discovered on the leaves of *D. alata* cultivar Da56 were morphologically and genetically like the SGG group found in West African yam and were named *C. alatae* [47]. While some authors have used the name *C. alatae* as the causal agent of yam anthracnose [34,41,47,48], others [28] used the name *C. gloeosporioides*. Since the isolates described in this study are like those reported by Weir et al. [34] and Lin et al. [47] we refer to our isolates as *C. alatae* as described by Ntui et al. [2].

The identification of *C. alatae* based on cultural, and morphological characteristics alone is not satisfactory as it can be mixed up with other species within the genus, most especially *C. Acutatum* [49, 50]. Moreover, different species of *Colletotrichum* can infect the same host and the foliage infection of *C. Acutatum* and *C. gloeosporioides* are difficult to differentiate in terms of their symptoms and cultural morphology [51, 52]; hence the need for the introduction of the molecular techniques for proper identification of the pathogen isolated in the present study. Polymerase Chain Reaction (PCR) amplification of the ITS region of *C. alatae* isolates in the present study gave amplicon size range of >500 bp which agrees with the findings of Raj et al. [53] who also observed band amplifications of above 500 bp when DNA from C*. gloeosporioides* isolates were amplified using the universal ITS primer pair. Shi et al*.* [51] however reported a slightly higher band size of 560 bp for *C. gloeosporioides* isolates. The variations in band sizes of the isolates in the present study indicate polymorphism in the isolated strains of *C. alatae* whose actual identities were further revealed in this study through sequencing of the ITS region genes. Comparative analyses of the sequences of the ITS region in the 6 isolates showed that isolates Ca5 and Ca14 were the most closely related with 81.4% nucleotide identity. Comparison of the ITS data from this study with *C. gloeosporioides* sequences published in the GenBank showed limited nucleotide identity (<75%) indicating that the isolates were quite distinct. These sequences will be deposited to NCBI as new isolate of *C. alatae.*

When yam landraces/accessions were challenged with *C*. *alatae* isolate Ca11, a high number of landraces were susceptible to the pathogen suggesting a high epidemic status of the sample area. The

presence of many susceptible yam accessions in this zone may be sustaining a high percentage of the pathogen reported in the forest and the southern guinea savanna zones [9] and may be responsible for a high severity (72%) level also documented in the forest zone [15] on some genotypes of *D. alata*. Extensive genetic diversity of *C*. *gloeosporioides* is visible at locations where their host populations originate and perpetuate compared to where such hosts were introduced. Nigeria, being a leading yam producing country in West Africa and, West Africa known as the yam belt region of the world is regarded as centre of diversity of a key yam pathogen [27]*.* The diversity of *C*. *alatae* is said to result from its high potential for gene flow; and its virulence anchored through the ease of recombination of its virulence alleles. The severity of *C. alatae* infection in an area is usually based on a host cultivar cum pathogen strain interaction and predicated on a combination of factors including genetic (its rapid evolution) and favourable environmental conditions [27].

**Conclusion**

With a high susceptibility status of the *D. alata* and *D. rotundata* landraces/accessions and the virulence of *C*. *alatae isolate* Ca11 identified in the Cross River yam farming areas, the propensity for large-scale epidemic looms. The outcome of this study is a pointer to the enormity of field losses that may have been incurred by farmers on a yearly basis over time and the impact on food security occasioned by anthracnose disease in this zone. These findings could serve as a lunch pad for plant breeders and molecular biologists, specifically, genomic specialists to genetically engineer *D. alata* and *D. rotundata* for resistance to anthracnose. A success in this direction will boost the confidence of yam farmers to venture more into yam farming even at mechanized levels. With this, more jobs will be created and the economy of the area and that of Cross River State, at large, will be stimulated and sustained.

**Acknowledgements**

This research was supported by the Nigerian Tertiary Education Trust Fund (TET Fund) grant

TETFUND/DR&D/CE/NRF/2016/STI/13/VOL.1	awarded	to	the	Department	of	Genetics	and

Biotechnology, University of Calabar, Calabar, Nigeria, for "Production of anthracnose-resistant yam

seedlings for use by Nigerian farmers.

**Conflict of interest**

The authors declare that no conflicts of interest exist.

**Author contributions**

E.E.E. and E.A.U. conceived the original concept. A.A.M and N.I.O carried out field survey and collected the samples. N.I.O., E.I.O. A.A.M and E.E.I. perform the experiments. V.O.N. analyzed the molecular data. V.O.N., A.A.M and E.A.U. wrote the manuscript. V.O.N. prepared the figures. All authors have read and approved the manuscript.

**References**

1. Fu, RHY, Kikuno H, Maruyama M. Research on yam production, marketing, and Future Prospects. IITA Research Guide 46, IITA Ibadan; 2011.
2. Ntui VO, Uyoh EA, Ita EE, Markson AA, Tripathi JN, Okon NI, et al. Strategies to combat the problems of yam anthracnose disease: Status and prospects. Mol Plant Pathol. 2021; 22:1302– 1314. [doi.org/10.1111/mpp.13107](https://doi.org/10.1111/mpp.13107)
3. Orkwor GC, Asiedu R, Ekanayake IJ. Food yams: Advances in research. Ibadan: IITA/NRCRI; 1998.
4. Markson AA, Omosun G, Madunagu BE, Amadioha AC, Wokocha R. Physicochemical alteration of tissues of white yam (*Dioscorea rotundata* poir) tubers incited by *Botryoiplodia theobromae* pat. Intl J Curr Res. 2010; 4: 055-061.
5. IITA (International Institute of Tropical Agriculture), Report, achievement, challenges, and prospects of yam production in Nigeria, IITA, Ibadan, Nigeria; 2013.
6. Bassey EE. Constraints and prospects of yam production in Nigeria. EJAFR 2017; 5(1), 55-64.
7. Burkill HM. The useful plants of Tropical Africa. Royal Botanical Gardens, Kew; 1985.
8. FAOSTAT, Food and Agricultural Organization of the United Nations. Rome, Italy. 2018 Yam production in Nigeria. Rome, Italy: FAO. Available from: www.fao.org. Accessed 11 May 2020.
9. Akem CN, Asiedu R. Distribution and severity of yam anthracnose in Nigeria. Pages 297-301 in: Root Crops for Food Security in Africa: Proc. Int. Soc. Trop. Root Crops (ISTRC)–Africa branch. M. O. Akoroda, ed. Kampala, Uganda; 1994.
10. Onyeka TJ, Petro D, Ano G, Etienne S, Rubens S. Resistance in Water Yam (*Dioscorea alata*) Cultivars in French West Indies to Anthracnose Disease Based on Tissue Culture-Derived Whole-Plant Assay. Plant Pathol. 2006; 55**:** 671-678. [doi.org/10.1111/j.1365-3059.2006.01436.x](https://doi.org/10.1111/j.1365-3059.2006.01436.x)
11. Akem CN. Yam Die-back and its principal cause in the yam belt of Nigeria. Pak J Biol Sci. 1999; 2 (4): 1106-1109. [doi.org/10.3923/pjbs.1999.1106.1109](https://dx.doi.org/10.3923/pjbs.1999.1106.1109)
12. CARDI. Caribbean Agricultural Research and Development Institute Annual Technical consumption of Nupe farmers of Niger State, central, Nigeria. Afri J Agri Res. 1995; 6(23): 5301-5313.
13. Simons, SA. Epidemiology and Control of Yam Anthracnose. Report of the Natural Resourse Institute, United Kingdom. 1993; pp. 44-68.
14. Green KR, Simons SA. Dead skin‟ on yams (*Dioscorea alata*) caused by *Colletotrichum gloeosporioides*. Plant Pathol. 1995; 43: 1062-1065. [doi.org/10.1111/j.1365-3059.1994.tb01660.x](https://doi.org/10.1111/j.1365-3059.1994.tb01660.x)
15. Egesi CN, Onyeka TJ, Asiedu R. Severity of anthracnose and virus diseases of water yam (*Dioscorea alata* L.) in Nigeria I: Effects of yam genotype and date of planting. Crop Prot. 2007; 26:1259-1265. [doi.org/10.1016/j.cropro.2006.10.025](https://doi.org/10.1016/j.cropro.2006.10.025)
16. Bem AA, Terna TP, Iyoula FI, Waya JI, Orpin JB, Manir N. Preliminary Studies on production and partial purification of toxins associated with black tar disease of yam (*Dioscorea Species*) In Makurdi, Benue State, Nigeria. IOSR-JESTFT. 2013; 3(3): 85-89. [doi.org/10.9790/2402-0338589](http://dx.doi.org/10.9790/2402-0338589)
17. Oben JA, Egbe AE, Chuyong GB, Tabot PT. Diversity of Yam (*Dioscorea* spp*.*) Populations in Southwestern Region of Cameroon. Am J Life Sci. 2016; 4(6): 187-194. [doi.org/10.11648/j.ajls.20160406.17](http://dx.doi.org/10.11648/j.ajls.20160406.17)
18. Barnett HL, Hunter BB. Illustrated genera of imperfect fungi. St. Paul. Minnesota: The American Phytopathological Society. 1998.
19. Abang MM, Winter S, Green KR, Hoffmann P, Mignouna HD, Wolf GA. Molecular identification of *Colletotrichum gloeosporioides* causing yam anthracnose in Nigeria. Plant Pathol. 2002; 51: 63–71. [doi.org/10.1046/j.0032-0862.2001.00655.x](https://doi.org/10.1046/j.0032-0862.2001.00655.x)
20. Lodhi MA, Ye GN, Weeden NF, Reisch BI. A simple and efficient method for DNA extraction from grapevine cultivars and *Vitis* species. Plant Mol Biol Rep. 1994; 12(1): 6-13. [doi.org/10.1007/BF02668658](https://doi.org/10.1007/BF02668658)
21. Kolade OA, Oguntade O, Kumar L. Screening for resistance to Yam anthracnose disease. Viology/Germplasm Health unit, IITA Ibadan. 2018.
22. Popoola AR, Adedibu BO, Ganiyu SA. Rapid assessment of resistance to tissue- cultured water yam (*Dioscorea alata*) and white yam (*Dioscorea rotundata*) to anthracnose (*Colletotrichum*

| *gloeosporioides* | Penz.). | Arch Phytopathol Pflanzenschutz. | 2013; | 46: | 663– | 669. |
| --- | --- | --- | --- | --- | --- | --- |
| [doi.org/10.1080/03235408.2012.749701](http://dx.doi.org/10.1080/03235408.2012.749701) | | |  |  |  |  |

23. Shivanna MB, Mallikarjunaswamy GE. Fungal diseases and their effect on phytochemical constituents of medicinally important species in Bhadra Wildlife Sanctuary, Karnataka, India. Indian Phytopathol. 2009; 62: 37-43.
24. Then PP, Jeewon R, Hyde KD, Pongsupasamit S, Mongkolporn O, Taylor PWJ. Characterization and pathogenicity of *Colletotrichum* spp. associated with anthracnose on chilli (*Capsicum* spp) in Thailand. Plant Pathol. 2008; 57: 562-572. [doi.org/10.1111/j.1365-3059.2007.01782.x](https://doi.org/10.1111/j.1365-3059.2007.01782.x)
25. Abera A, Lemessa F, Adunga G. Phenotypic characteristics of *Colletotrichum* species associated with mango (*Mangifera Indica* L.) in southwest Ethiopia. Food Sci Qual Manag. 2015; 46: 9-18.
26. Ayodele MA, Asiedu R. Field symptoms and laboratory of anthracnose diseases of *D. alata*. IITA. 2004; 16 pages.
27. Abang MM, Winter S, Hodeba D, Mignouna K, Green R, Asiedu R. Molecular taxonomic, epidemiological and population genetic approaches to understanding yam anthracnose disease. Afri J Biotechnol. 2003; 2(12): 486-496. [doi.org/10.5897/AJB2003.000-1098](http://dx.doi.org/10.5897/AJB2003.000-1098)
28. Kwodaga JK, Sowley ENK, Badii BK. Morphological and molecular characterisation of *Colletotrichum gloeosporioides* (Penz) isolates obtained from *Dioscorea rotundata* (Poir). Afri J Biotechnol. 2020; 19(5): 231-239. [doi.org/10.5897/AJB2020.17116](http://dx.doi.org/10.5897/AJB2020.17116)
29. Udhayakumar R, Usharani S, Muthukumar A. Pathogenicity variation, morphological and cultural characteristic of *Colletotrichum gloeosporioides* isolates. Plant Arch. 2019; 19(1): 425-430.
30. Zippora A, Kofi, BA, Emmanuel A, David A, Amarfo E. Variability of *Colletotrichum gloeosporioides* isolates the causal agent of anthracnose disease of cassava and yam plants in Ghana. Intl J Phytopathol. 2016; 05 (01): 01-09. [doi.org/10.33687/phytopath.005.01.1192](http://dx.doi.org/10.33687/phytopath.005.01.1192)
31. Martínez EP, Hío JC, Osorio JA, Torres MF. Identification of *Colletotrichum* species causing anthracnose on Tahiti Lime, Tree Tomato and Mango. Agron Colomb. 2009; 27 (2): 211-218.
32. Yee MF, Sariah M. Comparative Morphology and Characterization of *Colletotrichum* isolates Occurring on Cocoa in Malaysia. Pertanika J Trop Agric Sci. 1993; 16(1): 45-51.
33. Fokunang CN, Dixon AGO, Akem CN, Ikotun T. Cultural, morphological, and pathogenic variability in *Colletotrichum gloeosporioides* f. sp. *Manihotis* isolates from cassava (*Manihot esculenta*) in Nigeria. Pak J Biol Sci. 2000; 3 (4): 542-546. doi.org/10.3923/pjbs.2000.542.546
34. Weir BS, Johnston PS, Damm U. The *Colletotrichum gloeosporioides* species complex. Stud Mycol. 2012; 73: 115–180. [doi.org/10.3114/sim0011](https://doi.org/10.3114/sim0011)
35. Nwankiti AO, Okpala EU. Anthracnose of Water Yam in Nigeria. In: Tropical Root Crops:Research Strategies for the 1980s, Terry, E.R., K.A.Oduro and F. Caveness (Eds.)., IDRC., Ottawa, Ontario. 1981; pp: 208.
36. Guarnaccia V, Gilardi G, Martino I, Garibaldi A. Gullino ML. Species Diversity in *Colletotrichum* Causing Anthracnose of Aromatic and Ornamental Lamiaceae in Italy. Agronomy. 2019; 9: 613. [doi.org/10.3390/agronomy9100613](https://doi.org/10.3390/agronomy9100613)
37. Khodadadi F, González JB, Martin PL, Giroux E, Bilodeau GJ, Kari A. et al. Identification and characterization of *Colletotrichum* species causing apple bitter rot in New York and description of *C. noveboracense* sp. nov. Sci Rep. 2020; 10:11043. [doi.org/10.1038/s41598-020-66761-9](https://doi.org/10.1038/s41598-020-66761-9)
38. Widodo & Hidayat, S. H. Identification of *Colletotrichum* species associated with chili anthracnose in Indonesia by morphological characteristics and species-specific primers. Asian J Plant Pathol. 2018; 12: 7-15. [doi.org/10.3923/ajppaj.2018.7.15](https://dx.doi.org/10.3923/ajppaj.2018.7.15)
39. Martinez-Culebras P, Barrio E, Garcia, MD, Querol A. Identification of *Colletotrichum* species responsible for anthracnose of strawberry based on the internal transcribed spacers of the ribosomal region. FEMS Microbiol Lett 2000; 189: 97-101. [doi.org/10.1016/S0378-1097(00)00260-3](http://dx.doi.org/10.1016/S0378-1097(00)00260-3)
40. Nwadili CO, Augusto J, Bhattacharjee R, Atehnkeng J, Antonio L-M, Onyeka TJ. et al. Comparative Reliability of Screening Parameters for Anthracnose Resistance in Water Yam (*Dioscorea alata*). Plant Dis. 2017; 101, 209-216. [doi.org/10.1094/PDIS-06-16-0924-RE](https://doi.org/10.1094/PDIS-06-16-0924-RE)
41. Jayawardena RS, Hyde KD, Damm U, Cai L, Liu M, Li X. et al. Notes on currently accepted species of *Colletotrichum.* Mycosphere. 2016; 7(8): 1192-1260. doi.org/ 10.5943/mycosphere/si/2c/9
42. Achar KGS, Vasanthakumari MM, Mallikarjunaswamy PMG, Shivanna MB. Prevalence and severity of anthracnose of yam (*Dioscorea alata* and *D. bulbifera*) caused by *Colletotrichum gloeosporioides* in Bhadra Wildlife Sanctuary in Karnataka. J Mycol Plant Pathol. 2013; 43(3): 282-290.
43. Cannon PF, Damm U, Johnston PR, Weir BS. *Colletotrichum*–status and future directions. Stud Mycol. 2012; 73: 181–213. [doi.org/10.3114/sim0014](https://dx.doi.org/10.3114%2Fsim0014)
44. Sutton BC. The genus *Glomerella* and its anamorph *Colletotrichum*. In: *Colletotrichum*: Biology, Pathology and Control (Bailey JA, Jeger MJ, eds). CABI, Wallingford, UK. 1992; pp. 1–26.
45. Hyde KD, Cai L, Cannon PF, Crouch JA, Crous PW, Damm U. et al. *Colletotrichum*- names in current use. Fungal Divers. 2009; 39: 147–182. doi.org/fdp/sfdp/FD39-7
46. Winch JE, Newhook FJG, Jackson VH, Cole JS. Studies of *Colletotrichum gloeosporioides* disease on Yam (*Dioscorea alata* L.) in Solomon Islands. Plant Pathol. 1984; 33: 467–477. [doi.org/10.1111/j.1365-3059.1984.tb02870.x](http://dx.doi.org/10.1111/j.1365-3059.1984.tb02870.x)
47. Lin CH, Wu WQ, Liao XM, Liu WB, Miao WG, Zheng FG. First report of leaf anthracnose caused by *Colletotrichum alatae* on water yam (*Dioscorea alata*) in China. Plant Dis. 2018;
    102: 248. [doi.org/10.1094/PDIS-07-17-0979-PDN](https://doi.org/10.1094/PDIS-07-17-0979-PDN)
48. Bhunjun CS, Phukhamsakda C, Jayawardena RS, Jeewon R, Promputtha T, Hyde KD. Investigating species boundaries in *Colletotrichum*. Fungal Divers. 202; 107: 107– 127. [doi.org/10.1007/s13225-021-00471-z](https://doi.org/10.1007/s13225-021-00471-z)
49. Chowdappa P, Kumar SM. Existence of two genetically distinct populations of C*oolletotrichum gloeosporioides* Penz in mango from India. Pest manag Hort Ecosys. 2012.
    18: 161-170.
50. Reddy PP. Plant Protection in tropical root and tuber crops. India: Springer.2005.
51. Shi A, Kantartzi SK, Mmbaga MT, Chen P, Mrema F, Nnodu E. PCR- based markers for detection of *Colletotrichum acutatum* and *C. gloeosporioides* in flowering dogwood (*Cornus florida*). Australas Plant Pathol. 2008; 37: 65– 68. [doi.org/10.1071/AP07080](https://doi.org/10.1071/AP07080)
52. Serra IMRS, Menezes M, Coelho RSB, Ferraz GMG, Montarroyos AVV, Martins LSS. Molecular analysis in the differentiation of *Colletotrichum gloeosporioides* isolates from the cashew and mango trees. Braz Arch Biol Technol. 2011; 54: 1099– 1108. [doi.org/10.1590/S1516-89132011000600004](http://dx.doi.org/10.1590/S1516-89132011000600004)
53. Raj M, Jeeva, ML, Nath VS, Sankar S, Vidhyadharan P, Archana P. et al. A highly sensitive nested-PCR method using a single closed tube for the detection of *Colletotrichum gloeosporioides* causing greater yam anthracnose. J Root Crops. 2013; 39(2),163-167. [doi.org/10.1007/s12033-012-9496-9](https://doi.org/10.1007/s12033-012-9496-9)

**Table 1. Locations where yam farms were surveyed, and symptomatic leaves collected.**

|  | **S/N** | **Sampling Areas** | **Farm locations** |
| --- | --- | --- | --- |
|  |  |  |  |
|  | 1. | Obubra | Ovonum |
|  |  |  | Ofodua |
|  |  |  | Ochon, |
|  |  |  | CrutechObubra campus |
|  | 2. | Yakurr | Ugep Town |
|  |  |  | Ntankpo |
|  |  |  | Convent villages |
|  | 3. | Ogoja | Ndok |
|  |  |  | Mbok |
|  |  |  | Ukpe |
|  |  |  | EgojaNdim |
|  |  |  | Ekajuk |
|  |  |  | Okundi |
|  | 4. | Ikom | Nde |
|  |  |  | Ikom town |
|  |  |  | Edor |
|  |  |  | Okuni |
|  |  |  | Nkonfab |
|  |  |  | Alise |
|  | 5. | Akpabuyo | Akansoko |
|  | 6. | Calabar South | University of Calabar farms |
|  |  |  | New Airport farm |
|  |  |  |  |

**Table 2. *Dioscorea alata* and *Dioscorea rotundata* yam Accession/landraces and collection locations.**

|  | **Code** | **Local name** | **Status** | **Species** | **Location** |
| --- | --- | --- | --- | --- | --- |
|  |  |  |  |  |  |
|  | TDa 1100193 | - | Accession | *D. alata* | NCRI |
|  | TDa 1100010 | - | Accession | *D. alata* | NCRI |
|  | TDa 07100154 | - | Accession | *D. alata* | NCRI |
|  | TDa 1100432 | - | Accession | *D. alata* | NCRI |
|  | CA5 | Mkpasipiba | Landrace | *D. alata* | Calabar |
|  | CA6 | Ebeghe | Landrace | *D. alata* | Akpabuyo |
|  | CA7 | Efut | Landrace | *D. alata* | Akpabuyo |
|  | CA8 | Oboneje | Landrace | *D. alata* | Obubra |
|  | CA9 | Olele | Landrace | *D. alata* | Obubra |
|  | CA10 | Akabriinyang | Landrace | *D. alata* | Akpabuyo |
|  | CA11 | Ekautat | Landrace | *D. alata* | Akpabuyo |
|  | CA12 | Ebeghudukikot | Landrace | *D. alata* | Akpabuyo |
|  | CA13 | Obana | Landrace | *D. alata* | Yala |
|  | CA14 | Obunaonhlor | Landrace | *D. alata* | Yala |
|  | CA15 | Obunaolieyi | Landrace | *D. alata* | Yala |
|  | CA16 | Ogi 1 | Landrace | *D. alata* | Ishiagu |
|  | NA17 | Okpolukata | Landrace | *D. alata* | Yala |
|  | CA18 | Obana 2 | Landrace | *D. alata* | Yala |
|  | CA19 | Obunaigele | Landrace | *D. alata* | Yala |
|  | CA20 | Obunaochokpa | Landrace | *D. alata* | Yala |
|  | CR1 | Ogboja | Landrace | *D. rotundata* | Obubra |
|  | ER2 | Agbaocha | Landrace | *D. rotundata* | Ishiagu |
|  | ER3 | Orume | Landrace | *D. rotundata* | Ishiagu |
|  | ER4 | Iguma | Landrace | *D. rotundata* | Ishiagu |
|  | ER5 | Nka | Landrace | *D. rotundata* | Ishiagu |
|  | BR6 | Tiv yam | Landrace | *D. rotundata* | Benue |
|  | ER7 | Obiauturugo | Landrace | *D. rotundata* | Ishiagu |
|  | CR8 | Obubra | Landrace | *D. rotundata* | Obubra |
|  | TDr 11100873 | - | Accession | *D. rotundata* | NCRI |
|  | TDr 1000006 | - | Accession | *D. rotundata* | NCRI |
|  | CR11 | Ajaba | Landrace | *D. rotundata* | Yala |
|  | CR12 | Fakita | Landrace | *D. rotundata* | Yala |
|  | CR13 | Ijibo | Landrace | *D. rotundata* | Yala |
|  |  |  |  |  |  |

**Table 3. Primers designed, synthesized and used for identification of Anthracnose isolates.**

|  | Primer number | Primer name | Direction | Sequence |
| --- | --- | --- | --- | --- |
|  |  |  |  |  |
|  | P1 | YamCgITS1 | F | CCAGCGGAGGGATCATTACT |
|  |  |  | R | AAGTTCAGCGGGTATTCCTACC |
|  | P2 | YamCgITS2 | F | CCTGCGGAGGGATCATTACT |
|  |  |  | R | AAGTTCAGCGGGTATTCCTACC |
|  | P3 | YamCgITS3 | F | CCTGCGGCGGGATCATTACT |
|  |  |  | R | AAGTTCAGCGGGTATTCCTACC |
|  |  |  |  |  |

**Table 4. Cultural characteristics of *Colletotrichum alatae* isolates.**

| ***Colletotrichum*** | **Mycelia colour** | **Growth** | **Nature of** | **Colour on** | **Speed of growth** |  | **Mycelia growth** |
| --- | --- | --- | --- | --- | --- | --- | --- |
| ***alatae* isolates** |  | **Pattern** | **mycelia** | **reverse** |  |  | **rate** |
|  |  |  |  | **side** |  |  |  |
| Ca5 | Whitish/Black | Large | Cottony | yellow | Cover Petri dish in 7 | | 3.15 |
|  |  | concentric |  |  | days |  |  |
|  |  | ring |  |  |  |  |  |
| Ca11 | Light pink to | Small | Cottony | Light pink | Covered less than 2/3 | | 1.69 |
|  | Orange | concentric |  |  | of Petri dish in 10 days | |  |
|  |  | ring |  |  |  |  |  |
| Ca14 | Dark pink | Circular | Cottony | Light pink | Covered less than 2/3 | | 1.84 |
|  |  | growth |  |  | of Petri dish in 10 days | |  |
| Ca16 | Whitish to Black | Plain | Wooly | Dark Grey | Covered Petri dish in 3 | | 3.92 |
|  | Fast-growing |  |  |  | days |  |  |
|  | grey (FGG) |  |  |  |  |  |  |
| Ca22 | Brownish black | Large | Cottony | Light grey | Covered Petri dish in 9 | | 2.32 |
|  |  | concentric |  |  | days |  |  |
|  |  | ring |  |  |  |  |  |
| Ca24 | White/grey | Large | Cottony | Grey | Covered Petri dish in 8 | | 2.85 |
|  | Fast-growing | concentric |  |  | days |  |  |
|  | grey (FGG) | ring |  |  |  |  |  |
| Ca32 | Creamy/light | Small | Velvety | white | almost Covered | Petri | 2.74 |
|  | pink | concentric | cottony |  | dish in 10 days |  |  |
|  | Fast-growing | ring |  |  |  |  |  |
|  | salmond (FGS) |  |  |  |  |  |  |
| Ca34 | white | large | Cottony | white | Covered 2/3 of | Petri | 2.1 |
|  |  | concentric |  |  | dish in 10 days |  |  |
|  |  | rings |  |  |  |  |  |

**Table 5. Partial nucleotide sequences of ITS-rRNA of *Colletotrichum alatae* isolates.**

| **Sample ID** | **Sequences** |
| --- | --- |
| Ca5 | CCTGCGGAGGGATCATTACTGAGTTTACAACTCCCAAACCCAATGTGAACCATACCAAACTGTTGCCTCGCC |
|  | GGGGTCACGCCCCGGGTGCGTCGCAGCCCCGGAACCAGGCGCCCGCCGGAGGGACCAACCAAACTCTTTAC |
|  | TGTAGTCCCCTCGCGGACGTTATTTCTTACAGCTCTGAGCAAAAATTCAAAATGAATCAAAACTTTCAACAA |
|  | CGGATCTCTTGGTTCTGGCATCGATGAAGAACGCAGCGAAATGCGATAAGTAATGTGAATTGCAGAATTCA |
|  | GTGAATCATCGAATCTTTGAACGCACATTGCGCCCGCCAGTATTCTGGCGGGCATCCCTGTCCGAGCGTCAT |
|  | TTCAACCCTCGAACCCCTCCGGGGGGTCGGCGTTGGGGATCGGGAACCCCTCAGACGGGATCCCGGCCCCG |
|  | AAATACAGTGGCGGTCTCGCCGCAGCCTCTCCTGCGCAGTAGTTTGCACAACTCGCACCGGGAGCGCGGCG |
|  | CGTCGCAGCGTCCGTAAAACACCACAACTACTGAAATGTTGACCTCGGATACAGTAGGAATACCCGCTGAA |
|  | CTTG |
| Ca11 | CCTGCGGAGGGATCATTACTGAGGGGGGTTACGGGGCTTGACTCTCCCACCCTTTGTGACGTACCTCTGTTG |
|  | CTTTGGCGGCTCCGGCCGCCAAAGGACCTTCAAACTCCAGTCAGTAAACGCAGACGTCTGATAAACAAGTT |
|  | AATAAACTAAAACTTTCAACAACGGATCTCTTGGTTCTGGCATCGATGAAGAACGCAGCGAAATGCGATAA |
|  | GTAATGTGAATTGCAGAATTCAGTGAATCATCGAATCTTTGAACGCACATTGCGCCCCTTGGTATTCCGGGG |
|  | GGCATGCCTGTTCGAGCGTCATTACAACCCTCAAGCTCTGCTTGGAATTGGGCACCGTCCTCACTGCGGACG |
|  | CGCCTCAAAGACCTCGGCGGTGGCTGTTCAGCCCTCAAGCGTAGTAGAATACACCTCGCTTTGGAGCGGTTG |
|  | GCGTCGCCCGCCGGACGAACCTTCTGAACTTTTCTCAAGGTTGACCTCGGATCAGGTAGGAATACCCGCCTG |
|  | AACTTA |
| Ca14 | CCTGCGGAGGGATCATTACTAGGCGACTTCAAAACCCCCAATGTGACGTTACCAATCTGTTGCCCTCGGCCG |
|  | GGATTCTCTGCCCCGGGCGCGTCGCAGCCACGGAATCAACATGGCGCCCGCCGGAGGACCAACTCAAACTC |
|  | TTTTTTCTCTCCGTCGCGGCTTCCGTCGCGGCTCTGTTTTACCTTTGCTCTGAGCCTTTCTCGGCGACCCTAGC |
|  | GGGCGTCTCGAAAATGAATCAAAACTTTCAACAACGGATCTCTTGGTTCTGGCATCGATGAAGAACGCAGC |
|  | GAAATGCGATAAGTAATGTGAATTGCAGAATTCAGTGAATCATCGAATCTTTGAACGCACATTGCGCCCGCC |
|  | AGTATTCTGGCGGGCATGCCTGTCCGAGCGTCATTTCAACCCTCGAACCCCTCCGGGGGGTCGGCGTTGGGG |
|  | ATCGGCCCCTCACCGAGGCCGCCCCCGAAAATACAGTGGCGGTCTCGCCGCAGCCCTCTCCTGCGCAGTAGT |
|  | TTGCACACTCGCACCGGGGAGCGCGGCGCGGAACACACCCGTAAAACACCCCAAACTCTGAAATGTTGACC |
|  | TCGGGATCAGGAAGGAATACCCCCGCTGAACTTA |
| Ca24 | CATGCGGAGGGATCATTACTGAGTTTGGCATATCGGGCACCTCCACCCGTGCTTACCGTACCCTGTTGCTTC |
|  | GGCGGGCCCGCCTTCGGGCGGCCCGGGGCCTGCCCCCGGGACCGCGCCCGCCGGATACCCCAATGGAACAC |
|  | TGTCTGAAAGCGTGCAGTCTGAGTCGATTGATACCAATCAATCAAAACTTTCAACAATGGATCTCTTGGTTC |
|  | CGGCATCGATGAAGAACGCAACGAAATGCGATAACTAATGTGAATTGCAGAATTCAATGAATCATCGAGTC |
|  | TTTGAACGCACATTGCGCCCCCTGGTATTCCGGGGGGCATGCCTGTCCGAGCGTCATTTCTCCCCTCCAGCCC |
|  | CGCTGGTTGTTGGGCCGCGCCCCCCCGGGGGCGGGCCTCGAGAGAAACGGCGGCACCGTCCGGTCCTCGAG |
|  | CGTATGGGGCTCTGTCACCCGCTCTATGGGCCCGGCCGGGGCTTGCCTCGACCCCCAATCTTCTCAGATTGA |
|  | CCTCGGATCAGGTATGAATACCGTCTGAACTTGGATCAGATTACGCTAAGATCTTCGTCCGCTGCGGGGTAA |
|  | AGTAAAACGTGGAGGGGGAAGAGGGAGAGCAGGAG |
| Ca32 | CATTCTTGCGGAGGGTCCATTACTGAGAGAGGATCATGTAGTGCACGTCCACCATTGTTTACTAACGTAGTG |
|  | CTGCTTGGGTAGGCCTGGTCTGGCGTGCTGGGGGGCCGTCACAGCCCCGCCGGAGAGTGCCTGCCGACTGCC |
|  | TAAACCTCAAAATCTTTAACCAAACGTGTCTTTGTCTGAATAAACGTCTTTAATAAAAGCAAAACTTTCAAC |
|  | AACAGATCTCTTGGTTCTGGCATCGATGAAGAACGCAGCGAAATGCGATAAGAAATGCGAATTGCAGAATT |
|  | CTTTTGAGTCATCGAATCTTTGAACGCACATTGGGCCCTTTGGTATTCCTAAGGGCATGCATGTTCGAGCGTC |
|  | ATTTTCACTCCTCAAGCCCCCGGCTTGGTGTTGGAACGTTTGGTCCAGGGCCACCCCTGGACCCTATCCCAA |
|  | ATACAATACACGCGCTGGCTGTTGCACCCGCCGGTACACTGAACATCTTCACAGACCACGAAACCGGTCTCA |
|  | AGCGGTCGTACGGCCCCCCGGTCTACTCCTATATCTATCACAGAGATGACCCTCAGATCACGCAGGAATTAC |
|  | CGCCTGAACTTA |
| Ca34 | AAGTGGAAGAGATCATTACTGAGTTTAAAAAGTAGGGCTTCGTGCTGGCTACCACTTGCATGCTTCTGTGGA |
|  | GCAGCCCGGTCCCGTTCCTGGGGGGCGTCCCCCGCCAGGACCACTCACTCTGTTCTTTTTTAAACTTCTGCAA |

TCAAACCATAAAAAAATCAAAACTTTCATTTACGATCTCTTGGTTCGGGCTCTTGTTTCTGGGCAGCTGATGA ACATAAGTAATGTGAATTGCAAAATTCAAAGAATCAAATTCATCGTTGAACGCACATTGTTCCCGCCCATAG TGCCCTTTGGATATTCTGTACGAGCTGCCTGTTCACCCTCAATTTGTACTTGGTGTTTTTACTTGGGGTTGTTC GTCTTCCTCAAATTGATTGGCGGACACGTCCAGCTTCCATAGATTGGTAGTAAAACCTACTGGTACTGGCAA ATCATCACATTTTTGCCCTTGAAATCCAACTTATGACTGTTGACCATCAATACTGCATGAATACGCTTTGAAA CTTTAATCAGGTAGGAATACCCGCCTGAACTTAAATCTGTATTTATCGTCTTCTATGTGGTTTTTTTTATTTTG GGAGTTAACATAATTGCATCCGTGTGGGGTGTTTAA

**Table 6. Percentage nucleotide similarity among *Colletotrichum alatae* isolates from yam.**

| **Isolate/strain** | **KC010547** | **Ca5** | **Ca11** | **Ca14** | **Ca24** | **Ca32** | **Ca34** |
| --- | --- | --- | --- | --- | --- | --- | --- |
| KC010547* | 100 | 74.3 | 76.2 | 68.9 | 70.9 | 71.9 | 60.1 |
| Ca5 |  | 100 | 75.1 | 81.4 | 67.7 | 62.3 | 59.1 |
| Ca11 |  |  | 100 | 77.0 | 68.8 | 71.3 | 57.2 |
| Ca14 |  |  |  | 100 | 73.2 | 68.8 | 69.2 |
| Ca24 |  |  |  |  | 100 | 65.5 | 59.9 |
| Ca32 |  |  |  |  |  | 100 | 57.8 |
| Ca34 |  |  |  |  |  |  | 100 |

*Indicates the reference sequence from which the ITS primer 2 were designed

**Table 7. Disease incidence, disease severity score and resistance status in 20 Landraces of *Dioscorea alata* screened for anthracnose resistance.**

| **Yam landraces** | **No. of infected** | **Total No. of** | **Disease** | **Disease severity score** | **Rating*** |
| --- | --- | --- | --- | --- | --- |
|  | **leaves** | **leaves** | **incidence (%)** |  |  |
| TDa 1100193 | 14.3 | 76.6 | 18.66 | 1 | HR |
| TDa 1100010 | 21 | 70.3 | 29.87 | 2 | R |
| TDa 07100154 | 35.7 | 50.7 | 70.41 | 4 | S |
| TDa 1100432 | 57 | 82.3 | 69.25 | 4 | S |
| CA5 | 44.7 | 57.7 | 77.46 | 4 | S |
| CA6 | 38 | 55 | 69.25 | 4 | S |
| CA7 | 61 | 71 | 85.91 | 5 | HS |
| CA8 | 40 | 96 | 41.66 | 3 | MR |
| CA9 | 30 | 37 | 81.08 | 5 | HS |
| CA10 | 31 | 57.3 | 54.10 | 3 | MR |
| CA11 | 53.3 | 76 | 70.13 | 4 | S |
| CA12 | 68.3 | 94.3 | 72.42 | 4 | S |
| CA13 | 30 | 42 | 71.42 | 4 | S |
| CA14 | 26 | 121 | 21.48 | 2 | R |
| CA15 | 57.7 | 79.7 | 72.39 | 4 | S |
| CA16 | 65 | 103 | 63.10 | 4 | S |
| NA17 | 79 | 85 | 92.94 | 5 | HS |
| CA18 | 48 | 68.7 | 69.86 | 4 | S |
| CA19 | 54.3 | 90 | 60.33 | 3 | MR |
| CA20 | 53.3 | 95.7 | 56.69 | 3 | MR |

*Accessions or land races with a disease incidence of 0-20% were given a score of 1and considered as highly resistant

(HR), those with a disease incidence of 21-40% were given a score of 2 and regarded as resistant (R), those with a disease

incidence of 41-60% (3), and regarded as moderately resistant (MR), those with a disease incidence of 61-80% (4), and

regarded as susceptible (S), and those with a disease index of 81-100% (5) and regarded as highly susceptible (HS).

**Table 8. Disease incidence, infection indices and disease severity in 13 Landraces of *Dioscorea rotundata* screened for anthracnose resistance.**

|  | **Yam landraces** | **No. of infected** | **Total No. of** | **Disease** | **Disease severity** | **Rating*** |
| --- | --- | --- | --- | --- | --- | --- |
|  |  | **leaves** | **leaves** | **incidence (%)** | **(score)** |  |
|  | CR1 | 16 | 57.3 | 27.92 | 2 | R |
|  | ER2 | 23 | 69.3 | 3.18 | 2 | R |
|  | ER3 | 9.7 | 67 | 14.47 | 1 | HR |
|  | ER4 | 48 | 16.9 | 35.20 | 2 | R |
|  | ER5 | 8.3 | 65 | 12.76 | 1 | HR |
|  | BR6 | 8.7 | 91.3 | 9.52 | 1 | HR |
|  | ER7 | 40.7 | 45 | 90.44 | 5 | HS |
|  | CR8 | 20.7 | 26.7 | 77.52 | 4 | S |
|  | TDr 11100873 | 25.3 | 32.3 | 78.32 | 4 | S |
|  | TDr 1000006 | 9.7 | 51 | 19.01 | 1 | HR |
|  | CR11 | 15.3 | 46 | 33.26 | 2 | R |
|  | CR12 | 12.7 | 95 | 13.36 | 1 | HR |
|  | CR13 | 18.3 | 37.7 | 48.54 | 3 | MR |

*Accessions or land races with a disease incidence of 0-20% were given a score of 1and considered as highly resistant

(HR), those with a disease incidence of 21-40% were given a score of 2 and regarded as resistant (R), those with a disease

incidence of 41-60% (3) and regarded as moderately resistant (MR), those with a disease incidence of 61-80% (4) and

regarded as susceptible (S), and those with a disease index of 81-100% (5)and regarded as highly susceptible (HS).

**Figure legends**

**Fig. 1. a)** Map of Nigeria showing Cross rive state. **b)** Cross Rive State map showing locations where the farms were surveyed, and diseased leaf collected. The Nigeria and Cross River State blank maps

were retrieved from <https://www.mapsofworld.com/nigeria/nigeria-outline-map.html> [https://www.pngitem.com/middle/hhbRThx_map-of-cross-river-state-png-transparent-png/,](https://www.pngitem.com/middle/hhbRThx_map-of-cross-river-state-png-transparent-png/) respectively.

**Fig. 2.** Variation in appearance of anthracnose symptoms sampled on *D. alata* and *D. rotundata* in all the locations. Infected leaves sampled from Obubra sampling area (a-d), Yakurr (e-l), Ogoja (k-q); Ikom (r-s), Akpabuyo (t-u), and Calabar South (v-x). Arrow indicates symptoms.

**Fig. 3.** Morphological variability of *C. alatae* isolates obtained from the different locations (top row, upper side of the colony; bottom row, reverse side of the colony).

**Figure 4.** Photomicrographs showing growth pattern of fungi isolates from *D*. *alata* (a-e) and D. rotundata (f-h) at 10x. **a (Ca5),** one sided branching, non-branched tip, distance between branches ranges from 0.5 – 1.7cm, short bead-like hyphae with swollen tip. b (Ca11), alternate branching, non-branched tip, distance between branches ranges from 0.5 – 2.0cm, long cylindrical hyphae with tapering tip. **c (Ca14)** alternate branching, double branched tip, distance between branches ranging from 0.5 – 2.0cm, long branched thread-like hyphae with slightly swollen tip. d (Ca16) alternate branching with double branched tip, distance between branches ranges from 1.0 -2.5cm, long branched thread-like hyphae with swollen tip. e (Ca22) Alternate branching with non-branched tip, distance between branches ranging from 0.3 – 2.5cm, long branched cylindrical hyphae with tapering tip. f (Ca24), alternate branching, double branched at tip, distance between branches of about 0.5cm, short bead-like hyphae with slightly swollen tip. g (Ca32) alternating after two branches, triple branched at tip, distance ranging from 0.5 – 1.0, short bead-like branch with swollen tip. c (Ca34) alternating after two branches, multiple branched at tip, distance between branches ranging from 0.3 – 1.8cm, long bead-like hyphae with swollen tip.

**Fig. 5.** PCR amplification of ITS gene from 8 isolates of *Colletotrichum alatae.* a) PCR analysis using the three sets of primers. b) PCR analysis using primer set 2. 5,11*,*14, 16, 22 are isolates from *D. alata.* 24, 32 and 34 isolates *D. rotundata*. M, 1 kb DNA ladder, mx, DNA master mix.

**Fig. 6.** Phylogenetic tree showing relationships of closely related accessions with our isolates using Maximum Likelihood method and based on the ITS gene sequences. Ca5 to Ca34 *C. alatae* isolates obtained from this study.

Figure 1	[Click here to access/download;Figure;Figure 1.jpg](https://www.editorialmanager.com/pone/download.aspx?id=30352008&guid=be60e347-bdcc-479f-8f77-235e435515ea&scheme=1)

Figure 2	[Click here to access/download;Figure;Figure 2.tif](https://www.editorialmanager.com/pone/download.aspx?id=30352009&guid=4703255a-83eb-45a1-aec5-96e9b46d41a0&scheme=1)

Figure 3	[Click here to access/download;Figure;Figure 3.tif](https://www.editorialmanager.com/pone/download.aspx?id=30352016&guid=3fe24574-d54d-4c1b-8b99-6543084b3005&scheme=1)

Figure 4	[Click here to access/download;Figure;Figure 4.tif](https://www.editorialmanager.com/pone/download.aspx?id=30352027&guid=ca462df7-6742-4765-b234-6bf7bb793f67&scheme=1)

Figure 5	[Click here to access/download;Figure;Figure 5.jpg](https://www.editorialmanager.com/pone/download.aspx?id=30353287&guid=991a873b-cfff-4c34-abcb-21a346e8b314&scheme=1)

Th

Figure 6	[Click here to access/download;Figure;Figure 6.tif](https://www.editorialmanager.com/pone/download.aspx?id=30352030&guid=ad863b01-6ec5-4092-80a2-d8e8654c53cb&scheme=1)
