# Supplementary material for: Characterization of some fungal pathogens causing anthracnose disease on yam in Cross River State, Nigeria
Source: PLoS One. 2022 Jun 29;17(6):e0270601. doi: 10.1371/journal.pone.0270601 (PMC9242479; doi:10.1371/journal.pone.0270601)
Supplement: Attachment — Submitted filename: Response to Reviewers.docx [file pone.0270601.s005.docx]

**Response to Reviewers**

**To Handling Editor**

**Comment:** Please ensure that your manuscript meets PLOS ONE's style requirements, including those for file naming. The PLOS ONE style templates can be found at 
https://journals.plos.org/plosone/s/file?id=wjVg/PLOSOne_formatting_sample_main_body.pdf and 
<https://journals.plos.org/plosone/s/file?id=ba62/PLOSOne_formatting_sample_title_authors_affiliations.pdf>.

**Response:** Manuscript formatted accordingly

**Comment:** We note that you have provided funding information that is currently declared in your Funding Statement. However, funding information should not appear in the Acknowledgments section or other areas of your manuscript. We will only publish funding information present in the Funding Statement section of the online submission form. 
Please remove any funding-related text from the manuscript and let us know how you would like to update your Funding Statement. Currently, your Funding Statement reads as follows: 
 [This research was supported by the Nigerian Tertiary Education Trust Fund (TETFund) grant TETFUND/DR&D/CE/NRF/2016/STI/13/VOL.1 awarded to the Department of Genetics and Biotechnology, University of Calabar, Calabar, Nigeria, for "Production of anthracnose-resistant yam seedlings for use by Nigerian farmers.

**Response:** We have removed the funding statement from the manuscript. Please publish the funding statement as it is.

**Comment:** PLOS ONE now requires that authors provide the original uncropped and unadjusted images underlying all blot or gel results reported in a submission’s figures or Supporting Information files.

**Response:** We have included the original uncropped and unadjusted gel images.

**Comment:** Please include your full ethics statement in the ‘Methods’ section of your manuscript file. In your statement, please include the full name of the IRB or ethics committee who approved or waived your study, as well as whether or not you obtained informed written or verbal consent. If consent was waived for your study, please include this information in your statement as well.

**Response:** We have included ethical statement in materials and methods

**Comment:** We note that Figure 1 in your submission contain map images which may be copyrighted.

**Response:** Figure 1 has been deleted.

**To Reviewer 1**

**Comment:** What were the key findings from the characterization of the pathogen?

**Response:** Eight isolates, Ca5, Ca11, Ca14, Ca16, Ca22, Ca24, Ca32 and Ca34 of *C. alatae* were found to be associated with yam infection in Cross River State, with Ca11 as the most prevalent, occurring in all the locations. These isolates were classified into four forms which included the slow-growing grey (SGG), the fast-growing grey (FGG), the fast-growing salmon (FGS), and the fast-growing olive (FGO).

**Comments:** This section seems to be verbose and should be discussed briefly.

| **Response:** We have expunged some sentences  **Comment:** Is the pathogen infection issue in all form of Yam cultivars? And what about disease percent?  **Response:** This has been described in the introduction as follows: In Nigeria, *Colletotrichum* disease complex (commonly referred to as anthracnose or die-back) remains one of the most challenging and destructive diseases, causing heavy losses in yam. Anthracnose has been implicated in yam tuber yield losses ranging from 50 to 90% under favorable conditions for pathogen infection, establishment, and disease development  **Comment:** Objectives of this study not clearly mentioned  **Response:** We have rephrased the objective to: The objective of this study was to characterize and identify *Colletotrichum* isolates associated with yam anthracnose in Cross River State, Nigeria and to determine the relationship among them as well as their virulence.  **Comment:** The author must add the reference of isolation protocol  **Response:** The reference [16] has been added  **Comment:** Was the molecular characterization of any help in finding out any certain gene which can be used for further evaluating the infection capabilities of this pathogen?  **Response:** The molecular characterization was based on the ITS region. We did not do a whole genome sequence which would have revealed other genes which could be helpful in evaluating the infection capabilities of the isolates.  **Comments:** This section should be more clear and suggested for revision.  **Response:** We revised the DNA extraction protocol accordingly  **Comment:** Too much typo error. Use small case for chemicals name  **Response**: We have corrected all the typo errors.  **Comment:** During the characterization phase the phylogenetic analysis gave any other major infecting fungal species in closely clustered clade and what does it says overall for future of fungal infestation in Nigeria’s agriculture fields?  **Response:** Yes, there were some other *Colletotrichum* species found in same clade with some of our isolates, but quite distinct in terms of nucleotide identity. This however, indicates there could be a mixed infection of yam in the field. We have added this phrase in the discussion.  **Comment:** Did the author repeat this experiment for the confirmation of results?  **Response:** We did not repeat the experiment. This was because we also did whole plant bioassay. Besides, the pathogenicity test was done in very sterile condition without any contamination  **Comment:** Correct equation format suggested  **Response:** This has been corrected as suggested  **Comment:** Give specific name to the primer set rather than 1 or 2  **Response:** This has been corrected  **Comment**: This section (discussion) is too verbose which makes difficulty for the readers. The author suggested to concise it with necessary information and literature list.  **Response:** This has been done as suggested  **Comment:** What are ultimate domestic market and export market benefits of your studies?  **Response:** This study will be useful to yam breeders in Nigeria and elsewhere. Please see our conclusion.  **To Reviewer 2**  Abstract  **Comment:** The abstract is written well. However, some of the data are misleading in screening the landraces which are repeated esp . … 15.15% were moderately resistant (MS), which is wrongly represented. 18.18% were resistant while 18.18% were highly resistant (HR). The data is not clear.  **Response:** We have corrected the sentence to: Inoculation of 20 *D. alata* and 13 *D. rotundata* landraces with isolate Ca11, showed that 63.64% of the landraces were susceptible while 36.36% were resistant  Introduction:  **Comment:** Can be shortened and the importance of Yam can be reduced. Instead the authors can focus in the disease and the present control measures followed. Besides, the international status of the disease and pathogen can also be represented as described by other scientists.  **Response:** This has been done as suggested  Materials and Methods: Comment: Should be clear and concise and the methodology should be described. The cultural and morphological characters used for identification were not mentioned in this section. The type and methods of survey carried out can be elaborated. While using a scale, you can follow percent disease index rather than incidence. Any previous reference is there for disease scale. Why data were recorded 16 weeks post inoculation? Whether the symptoms appeared only by that time? How long the plants were maintained in the glass house. In soil or in pot culture?. The concentration (spores/ml) of the inoculum should be mentioned. Statistical analysis followed should be included. How many replications were made for the screening experiment. How many plants were used for landraces/accessions for screening?  **Response:** Please see the responses below, these have also been incorporated in the manuscript  Cultural and morphological characteristics by which the isolates were identified include mycelia colour, growth pattern, nature of mycelia and speed of growth in the Petri dish. All these characters were done through visual appreciation.   i) The sampling method used was cluster sampling. The local government areas were the clusters where farms were identified and sampled in selected villages (units) within the Local Government areas. Simple random sampling procedures were used in selecting the villages in each of the Local Government Areas. As such each of the villages had equal chances of being selected since all were known to be major yam-cultivating area. Hence six Local Government Areas (six clusters) were used and villages with history of yam cultivation were selected randomly to arrive at the villages sampled per Local Government Area. ii) Symptoms started appearing at 8 weeks post inoculation and progressed slowly, so we allowed the symptoms to develop fully before taking data at 16 weeks. iii) Plants were maintained for 6 months in bags. iv) Spore concentration used was 1.0 x 10^4^ spore/ml throughout the study. v) During field screening, three replicates were maintained. vi) 27 Landraces and 6 accessions were screened. Each was replicated thrice (3 plants per landrace/accession).     **Results** The result section can be reduced and at present it is elaborate. Some queries which are observed in the result are  **Comment:** The figures and tables can be reduced. Some tables can be deleted viz., Table 5 and Table 6. Table 3 can be given as a supplementary file. Similarly, Table 7 & 8 can be clubbed and the disease incidence and reaction can alone be presented as a single table.  **Response:** The figures have been reduced to 5. The number of Tables have been reduced to 4. We have deleted Table 5. We have taken Tables 3 and 6 to supplementary file as supplementary Tables 1 and 2. We combined Tables 7 and 8 as one Table which is now Table 4.  **Comment:** Whether rapid growth in plate is directly proportional to the virulence can be explained.  **Response**: The rapid growth on the plates does not directly transcribe to virulence as the isolate Ca 11 which is one of the slowest growing (Table 3) is the most virulent among the isolates studied.  **Comment:** Whether any specific difference in mycelial growth pattern was observed.  **Response:** Differences in mycelia growth pattern were also observed. This ranged from small to large concentric rings exhibiting mostly cottony growth mycelia (Table 3).  **Comment:** Whether Acervuli was observed in the isolate in the diseased specimens. You can document the data to see the difference.  **Response:** Other characteristics such as Acervuli were observed in the plates but not in infected specimens. These had dark spines (setae) at the edge of the structure and among the conidiophores (Fig.3j)  **Comment:** Any sexual fruiting body was observed in the cultures viz., Perithecia. Since it deals with differentiating Colletotrichum spp. these characters are important.  **Response:** Perithecia were not observed  **Comment:** No conidial photos of the isolates were presented.  **Response:** Photos of the two types of conidia encountered have been added (Fig. 3i)  **Comment:** Is there any variation of the hyaline, single celled conidia of C. alatae with respect to C. gloeosporioides observed as there exist variation among the species of Colletotrichum.  **Response:** there were no distinct differences in the conidial appearance in terms of size and shape. All were single -celled, oblong or cylindrical, broadly rounded at both ends and some slightly tapering to the base (Fig. 3i)  **Comment:** Amplification of ITS region cannot differentiate within species level. If there are any SCAR markers specific for C. gloeosporioides, it can be used to differentiate C. alatae.  **Response:** We also intended to use some molecular markers to confirm the identification of the isolates. However, the project ended and we did not have funds to continue with the work. We are however sourcing for funds, as soon as we get some funding we will do detail molecular analysis of the isolates, using some molecular markers.  **Comment:** Some RAPD and ISSR primers can be used to differentiate the isolates which can be used to identify the marker unique in C. alatae.  **Response:** As explained already, we intended to use some molecular markers to confirm the identification of the isolates. However, the project ended and we did not have funds to continue with the work. We are however sourcing for funds for another level of study on these isolates using these primers.  **Comment:** The accession number to the sequenced isolate can be given instead of the sequence.  Response: This has been done  **Comment:** The variation in the sequence with respect to C. gloeosporioides can be highlighted if the authors justify the portion of the genome which is variable.  **Response:** We have highlighted this in the text as follows: The 5.8s rRNA was the most conserved genomic region across the isolates and the C. *gloeosporioides* reference genome with minimal variations, whereas the ITS1 and ITS2 were the most diverse.  **Comment:** The sequence of CA11 presented in Table 5 if blasted in NCBI database does not any match to C. alatae. Rather it shows identity to Lasiodiplodia theobromae. Justify.  **Response:** We have justified this in the discussion as follows: Isolate Lt1 was the most virulent, but it was one of the slow growing types in the plates, indicating that rapid growth in the plate is not proportional to virulence. A blast of this isolate on NCBI shows <75% nucleotide identity with other *C. gloeosporioides*, and >97% nucleotide identity with many *Lasiodiplodia theobromae* isolates. *L. theobromae* is reported to be cosmopolitan in nature and has been reported to cause dieback infections on cash crops such as cocoa and yams [54]. This and the fact that the isolate clustered in the same clade with *L. theobromae* supported by high boostrap value (Fig. 5), confirmed its identity. Therefore, we named it *L. theobromae* isolate Lt1 with the accession number OM365423. When yam landraces/accessions were challenged with this isolate, a high number of landraces were susceptible to the pathogen suggesting *L. theobromae* infects yam. More studies are required to evaluate the prevalence of *L. theobromae* infection of yam in Nigeria*.*  **Comment:** Whether you have submitted the sequence in the NCBI or any other database and the accession number obtained is not clear.  **Response:** We have included a statement on this in the results as follows: The nucleotide sequences of the ITS region for six of the isolates were submitted to National Centre for Biotechnology Information (NCBI) database and were assigned the following accession numbers: OM365422 (Ca5), OM365423 (Lt1), OM365424 (Ca14), OM365425 (Ca24), OM365426 (Ca32) and OM427500 (Ca34).  **Comment:** Disease incidence of 41-60 % is given as moderately resistance which looks very high. If the plant is 60 % infected how can we place in the resistant category. Pl. refer the reference for the reaction scale.  **Response:** We have corrected this to show that disease indices above 40% are considered to be susceptible (Table 4)   Discussion **Comment:** It can be reduced to a certain extent and the main point alone can be discussed. However, the authors have substantially discussed the variation within the pathogen that may have arised due to recombination/gene flow.  **Response:** The discussion has been considerably reduced  Conclusion :  **Comment:** Conclusion should directly depict the results from the manuscript and its application in future can be highlighted.  **Response:** This has been corrected as suggested. |  |  |
| --- | --- | --- |
