# Supplementary material for: Characterization of some fungal pathogens causing anthracnose disease on yam in Cross River State, Nigeria
Source: PLoS One. 2022 Jun 29;17(6):e0270601. doi: 10.1371/journal.pone.0270601 (PMC9242479; doi:10.1371/journal.pone.0270601)
Supplement: Attachment — Submitted filename: Response to Reviewers.docx [file pone.0270601.s006.docx]

Response to comments

Reviewer #2: The authors have carried out all the corrections as suggested by the reviewer. Now the quality of the paper is improved. But there are many basic flaws which need to be addressed besides some minor corrections.
Minor corrections

Comment: Abstract: Spelling mistakes . Need to be carefully addressed throughout the manuscript. Lsidioplodia theobromae, (Lasiodiplodia theobromae).

Response: This has been corrected in the abstract and throughout the manuscript

Comment: In the third line include…In the present study, the pathogen…….

Response: The phrase has been added.

End of Introduction Proffer ….
The identity of the isolates in sequencing seems to be very less and should be clarified. Bec. < 75 % identity could not exist within a species. In that case, it should be a different genus.

Response: Although the isolates had < 75% identity, they were identified as C. alata based on their cultural and morphological characteristics, mycelial growth rate as well as the fact that they clustered with other Colletotrichum species.

Comment: None of the C. alatae isolates was in the same cluster with the reference sequence indicating that the isolates are distinct……Is not clear, If so how you claim it is C. alatae if it is distinct….Can be rewritten or reframed.

Response: The sentence has been rephrased to: None of the *C. alatae* isolates was in the same cluster with the reference sequence KC010547.1 but were found clustering with other *C. gloeosporioides.*

Note: The reference sequence was used to design primers. It could be that the primers did bind our isolates, but the nucleotide identity is not the same. That is why the isolates are clustering with other *C*. *gloeosporioides* and not with the reference sequence*.*

Major corrections

Comment: In the materials and methods in whole plant bioassay it is denotes as
Screening of yam for tolerance to C. alatae: Whole plant bioassay

Response: We have corrected this to: Screening of yam for tolerance to *Lasiodiplodia theobromae*: Whole plant bioassay

Comment: In the above title, you have mentioned tolerance to C. alatae. But challenged with Lasiosiplodia theobromae ……The yam plants were challenged with isolate Lt1 which was the most virulent according to the pathogenicity test. ……….Then the entire experiment goes wrong as it is with a different pathogen.

Response: We have modified the title to: Characterization of some fungal pathogens causing anthracnose disease on yam in Cross River State, Nigeria

Comments: Even though it is highly pathogenic, it is a different pathogen with different spore characters. In that case, the paper should address the importance of two pathogens and not only C. alatae. Bec. The title deals with C. alatae. But the landraces/accessions are screened for L. bromae. The authors will be confused with these statements. Substantiate.

Response: We have modified the title and addressed *Lasiodiplodia theobromae* in the manuscript

If Lt1 is used for screening the isolates how will you claim it is resistant to Colletotrichum spp? In that case, even the scale used should have a reference as it is general or specific for Colletotrichum pathogens.

Response: We have modified the section to: Screening *D. alata* and *D. rotundata* for resistance to *Lasiodiplodia theobromae* in the screen house. The scale used is general for classification of disease resistance

Comment: In the table 4 in the legends it is still indicated that 41- 60 % is moderately resistant but in response to reviewers it is claimed that > 40 % is considered as susceptible. Clarify.

Response: We have corrected the legend as follows: Accessions or land races with a disease incidence of 0-20% were given a score of 1and considered as highly resistant (HR), those with a disease incidence of 21-40% were given a score of 2 and regarded as resistant (R), those with a disease incidence of 41-60% (3), and regarded as moderately susceptible (MS), those with a disease incidence of 61-80% (4), and regarded as susceptible (S), and those with a disease incidence of 81-100% (5), and regarded as highly susceptible (HS). In general, indices >40% are susceptible.
